# Something has to give: scaling combinatorial computing by biological agents exploring physical networks encoding NP-complete problems

**DOI:** 10.1098/rsfs.2018.0034

**Published:** 2018-10-19

**Authors:** Falco C. M. J. M. van Delft, Giulia Ipolitti, Dan V. Nicolau, Ayyappasamy Sudalaiyadum Perumal, Ondřej Kašpar, Sara Kheireddine, Sebastian Wachsmann-Hogiu, Dan V. Nicolau

**Affiliations:** 1 Molecular Sense Ltd, Liverpool L36 8HT, UK; 2 Department of Bioengineering, McGill University, Montreal, Quebec, Canada H3A 0E9; 3 School of Mathematical Sciences, Queensland University of Technology, Brisbane, QLD 4000, Australia; 4 Department of Chemical Engineering, University of Chemistry and Technology, Prague, Technická 5, 166 28 Prague 6, Czech Republic

**Keywords:** network-based computation, bio-computation, combinatorial problems, NP-complete problems, hardware-embedded solutions, subset sum problem

## Abstract

On-chip network-based computation, using biological agents, is a new hardware-embedded approach which attempts to find solutions to combinatorial problems, in principle, in a shorter time than the fast, but sequential electronic computers. This analytical review starts by describing the underlying mathematical principles, presents several types of combinatorial (including NP-complete) problems and shows current implementations of proof of principle developments. Taking the subset sum problem as example for in-depth analysis, the review presents various options of computing agents, and compares several possible operation ‘run modes’ of network-based computer systems. Given the brute force approach of network-based systems for solving a problem of input size C, 2^C^ solutions must be visited. As this exponentially increasing workload needs to be distributed in space, time, and per computing agent, this review identifies the scaling-related key technological challenges in terms of chip fabrication, readout reliability and energy efficiency. The estimated computing time of massively parallel or combinatorially operating biological agents is then compared to that of electronic computers. Among future developments which could considerably improve network-based computing, labelling agents ‘on the fly’ and the readout of their travel history at network exits could offer promising avenues for finding hardware-embedded solutions to combinatorial problems.

## 1. Introduction

Many combinatorial problems of practical importance, including NP-complete problems, appear to require that an extremely large number of possible candidate solutions is explored in a brute-force manner in order to discover the actual solutions. Examples of such problems are the design and verification of circuits [1], the folding [2] and design [3] of proteins, optimal network routing [4], formal reasoning [5] and data clustering in complex networks [6]. When the size of these problems grows, the time required to find solutions on sequential computers grows exponentially. Consequently, solving these problems by any computer that performs computations sequentially, including electronic computers, requires unreasonable computing times, even for medium-sized problems, as implied by the NP-Hardness Assumption [7]. Therefore, to solve these problems in practice will require efficient parallel computation approaches [8], but those presently proposed raise various critical technical difficulties. For instance, DNA computing generates mathematical solutions by recombining DNA strands [9,10], or DNA-static [11] or -dynamic [12] nanostructures, but this approach requires impractically large amounts of DNA [13–16]. Quantum computing appears to be limited in scale by decoherence and by the small number of qubits that can be integrated [17]. Finally, microfluidics-based parallel computation [18] is difficult to scale up with the size of the problem due to the rapidly diverging physical size and complexity of the devices.

A recently proposed alternative, network-based computation [19], may be capable of overcoming some of these scalability problems. A network-based computing device comprises a network which is a physical embodiment of a graph representing an instance of a mathematical problem. The network-based computation consists of the directed movement of motile physical objects—computation agents—through the entries, conduits, nodes and exits of the computer network. The history of the positions of the exploring agents through the encoded network, if decoded, represents the solution to the mathematical problem. Consequently, DNA computing does not represent a subset of network-based computation, although it does attempt to solve an NP complete problem which does have a classical graph-based representation. The core concept of network-based computing is to map the set of all possible solutions of mathematical problems into physical structured pathways to encode the ‘content’ of the problems, and then to find the solution(s) by exploring these pathways using a large population of autonomous, self-propelled ‘agents’, such as molecular motor-driven cytoskeletal filaments [20], or microorganisms [19]. This approach was used to demonstrate the principle of solving in a combinatorial manner a small instance of an NP-complete problem, the subset sum problem (SSP) [20]. The estimated energetic efficiency in this computation approach also was orders of magnitude higher than that of electronic computers, suggesting that such a technology might circumvent the heat dissipation which is one of the limiting factors in developing ever-larger classical supercomputers [21].

Like other alternatives to sequential computing, network-based computation, in particular using biological agents, faces scalability issues of its own. Consequently, it is imperative to identify the engineering bottlenecks blocking the progress and explore possible avenues for alternative solutions. This methodological approach is expected to lead to the aggregation of a ‘road map’, similar to the one formally developed by the community of the semiconductor industry. To this end, this contribution maps the current state-of-the-art in network-based computation, with an emphasis on the use of biological agents, starting with the mathematical principles, comparing various types of computing agents, technological challenges related to fabrication and readout, and opportunities regarding energy efficiency. Drawing this all together, we attempt to identify the advancements in several ‘service technologies’ that are likely to be necessary for network computing with biological agents to become useful for real-world applications.

## 2. Network-based computing with agents

### 2.1. Network-based computing: concepts and tentative implementations

Supposing that specific NP-complete problems can be formulated as *graphs* [19], it is possible to translate these into *designs of physical networks*, i.e. graphs with dimensions, e.g. distances between vertices, widths of the lines connecting these vertices, etc. These designs then can be the basis of the fabrication of physical networks, such as microfluidic structures comprising channels, nodes, entries and exits. These devices are essentially *computer networks* that encode the NP-complete problem of interest, which then ‘waits’ to be solved through the stochastic exploration, in parallel, by a large number of independent agents which act as ‘*processors*’ (pseudo-central processing units (CPUs)), each searching independently for a solution, through the process of moving from one junction to another ‘downstream’ from the entry towards (one of) the exit(s). Essentially, the physical network is not the computer *per se*, but it is the physical input to calculations. Therefore, network-based computing combines the ‘hardware’ design of networks, encoding mathematical problems of interest (see electronic supplementary material, SI-1, for the mathematical formulation), with the ‘software’ or information-processing capacity of a population of agents freely and stochastically exploring this network in a combinatorial fashion.

This staged process, i.e. graph encoding a mathematical problem → design of a physical network → fabrication of a microfluidic device → massively parallel exploration of the network by a large number of agents, has been recently proposed [20] as a proof of principle for solving the SSP. However, this strategy is amenable to other graph-based formulations of NP-complete problems.

**Figure 1.**
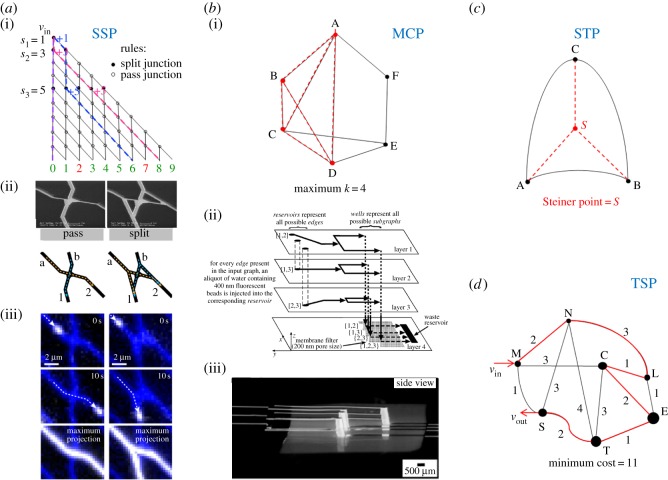
(*a*) Subset sum problem (SSP). (*a*(i)) Representation of a computation network for the subset sum of {1,3,5}. The agents start in the top left-hand corner. The junctions of the paths are: *filled circles*: SPLIT junctions where the agents have a 50% probability of continuing their straight path or to turn, or *empty circles:* PASS junctions where the agents always continue their straight path. Moving straight down at a split junction corresponds to *not* adding an integer to a running sum (purple example path). Moving diagonally down at a split junction corresponds to adding that integer (numbers 1 and 5 for the blue example path). The actual value of the integer potentially added at a SPLIT junction is determined by the number of rows of PASS junctions following that particular SPLIT junction (numbers indicated on the left of the paths). Green exit numbers represent sums for which a matching subset exists, and red numbers represent sums for which no matching subsets exist. (*a*(ii)) SEM graphs and schematic of pass (left) and split (right) junctions [20], where entrance and exit channels are labelled a and 2 respectively for agents travelling on diagonal paths, while entrance and exit channels for agents moving in a vertical path are labelled b and 1. The yellow dotted lines indicate diagonal paths and blue dotted lines indicate straight paths. (*a*(iii)) Fluorescence micrographs highlighting paths of moving microtubules across a pass (left) and a split (right) junction. Images in the third row show the maximum projection of agents in motion. Figures (*a*(ii)) and (*a*(iii)) are adapted from Nicolau *et al*. [20] (Copyright 2016) which also presents an animation detailing the function of the SSP computing principle. (*b*) Clique problem (CP). (*b*(i)) Maximum clique problem (MCP) computed on a given undirected graph *G* comprising nodes *n* = {*A*, *B*, *C*, *D*, *E*, *F*}. The maximum clique (highlighted in red) results to be of size *k* = 4 with vertices subset {*A*, *B*, *C*, *D*}. (*b*(ii)) Schematic of a four-layer microfluidic device used in solving an MCP for a graph having three vertices [18]. This three-dimensional microfluidic system has reservoirs—where a plug of fluorescent beads is injected—to represent all of the possible edges of a graph with three vertices, and wells—where the fluorescent beads are collected by a size filter sandwiched between the bottom and the top three layers—to represent all possible subgraphs of a three-vertex graph. The arrows in the schematic indicate directions of fluid flow; suction (house vacuum) is applied at the waste reservoir to drive fluid flow from the reservoirs representing edges to the waste reservoir. (*b*(iii)) Fluorescence photograph of the actual device for solving a three-vertex graph, viewed from the side. Figures (*b*(ii)) and (*b*(iii)) are adapted from Chiu *et al.* [18] (Copyright 2001 National Academy of Sciences, USA). (*c*) Steiner tree problem (STP). Out of the possible connection paths between three nodes on an undirected graph, *n* = {*A*, *B*, *C*} a single Steiner point, *S* joins the vertices with minimum distance. (*d*) Travelling salesman problem (TSP)*.* A representation of the TSP by an undirected graph with designated vertices *v*_in_ = *M* and *v*_out_ = *S*, for which the minimum cost Hamiltonian path is *M* → *N*, *N* → *L*, *L* → *C*, *C* → *E*, *E* → *T*, *T* → *S* with as total cost, *C* = 11.

Various implementations of network computing schemes encoding NP-complete problems, using various computational agents, have been attempted. Importantly, all reported implementations used solely the combinatorial run mode (see §2.2), i.e. a large number of agents exploring simultaneously a physical network encoding the mathematical problem. Some of these problems, and their implementation in proofs of principle devices, are reviewed (and presented in figure 1).

#### 2.1.1. Subset sum problem

The *subset sum problem*, a benchmark combinatorial problem [22], asks whether, given a set *S* = {*s*_1_, *s*_2_, …, *s_n_*} of *n* integers, there exists a subset of *S* whose elements sum to a target integer, *T* (figure 1*a*(i)). SSP has applications in various fields, especially when optimizing resource usage under constraints, and the ‘hardness’ of the problem is harnessed in certain cryptographic systems to generate encoded messages [23], due to its simple construction and resistance to quantum attacks [24]. Also, SSP, or its variant, the knapsack problem [25], finds applications in resource allocation for specialized producers, in efficient throughput and congestion allocations despite selfish users' behaviour, in the allocation of bandwidth in communication networks based on user requests, and in auctions. A recent review [26] provides an insightful discussion on existing and possible applications.

A methodology to solve the SSP that uses biological agents has been proposed [19] and recently demonstrated [20], using cytoskeletal filaments, i.e. actin filaments, or microtubules, propelled by protein molecular motors, i.e. myosin, or kinesin, respectively (figure 1*a*(ii,iii)). Interestingly, there have also been proposals for solving SSP by optical computing [27,28], but it was found that the energy required for large problems is prohibitive.

#### 2.1.2. Clique problem

The *clique problem* (CP) asks, for a network *G* of *n* vertices and *p* edges, if there are subsets of nodes with *k* vertices within *G* that have the property of all their members being completely connected to one another (‘cliques’). Several formulations of the problem exist, of which the maximum clique problem (MCP) is the best known. The MCP consists in listing all maximal cliques that cannot be enlarged by solving the decision problem on whether *G* contains a larger clique than the current size *k*. MCP asks to determine a complete subgraph of maximum cardinality, or maximum vertices [29]. Figure 1*b*(i) represents the MCP problem for a given graph *G* = (*n*, *p*) with *n* = {*A*, *B*, *C*, *D*, *E*, *F*}. A brute force algorithm exploring all possible solutions finds out that the set of vertices {*A*, *B*, *C*, *D*} is a maximum clique of *G*, and therefore that the maximum *k* = 4. MCP is notable for its relevance to a large number of applications, e.g. bio- and chemo-informatics, coding theory, economics, examination planning, financial networks, scheduling, signal transmission analysis, social network analysis, and wireless networks and telecommunications. A recent review [29] provides a comprehensive bibliography.

CP has been solved [18] by means of network computing using a multi-layered, three-dimensional microfluidics structure (figure 1*b*(ii),(iii)), which encodes the MCP for a simple graph with six vertices, explored by beads carried by fluid flow. While the calculation and the readout are done in parallel, the computing process is biased, as the beads will follow the lowest pressure lines in the flow, rather than independently explore the solution space. Also, the power required for pumping the fluid in microfluidic channels grows exponentially with the size of the problem, resulting in an unreasonable pressure build up [20]. The MCP has also been solved by DNA computing [15].

#### 2.1.3. Steiner tree problem

The *Steiner tree problem* (STP), and one of its special cases, the minimal Steiner tree problem (MSTP), asks, given a network *G* comprising *n* nodes and *p* edges, and a special subset of those edges (usually called terminals), for a tree that contains all these terminals (but which may include additional vertices) [30]. As with most NP-complete problems, there are a number of variants, but all ask, ultimately, for an optimal interconnect for a given set of objects, *subject* to a predefined objective function. Figure 1*c* represents the STP problem computed on an undirected graph *G* = (*n*, *p*) with *n* = {*A*, *B*, *C*}. An algorithm searching for the minimum cost connected set that joins all the given nodes eventually decides that the solution lies at a single Steiner point *S*. STP is relevant to many applications, e.g. VLSI physical design, FPGA routing placement, telecommunication network design, keyword-based selection of relational databases, data-centric routing in wireless sensor networks, multicast packing, network topology control, and access strategies design for ISP networks. A recent report [31] provides a detailed bibliography regarding these applications.

STP, in particular the MSTP, has been solved [31,32] using a slime mould (*Physarum polycephalum*) inspired algorithm. It should be noted, however, that solving optimization problems, such as the STP and the travelling salesman problem (see below) using slime moulds does not perfectly fit the definition of network computing with agents, because the edges of the graph are not pre-determined, and because the slime mould represents a collection of agents, i.e. tubular elements, which do not operate independently.

#### 2.1.4. Travelling salesman problem

The travelling salesman problem (TSP) is one of the best known NP-complete problems. Given a graph such that cities are vertices and the distances between them correspond to the graph's weighted edges, the problem asks for the shortest route (or another performance criterion, e.g. lowest travelling cost) between ‘cities’ under the condition that each valid route visits each ‘city’ only once. In other words, the problem asks to find the Hamiltonian cycle, being the path that visits every node once, at minimum cost. Figure 1*d* shows an undirected graph *G* = (*n*, *p*) with *n* nodes being the set of cities and *p* edges being the possible paths. For each new node visited, the total cost *C* is incremented by the weight, equivalent to the distance travelled to visit the new node. The algorithm computes by brute force all possible tours under the initial conditions of start, *v*_in_ = *M* and finish point, *v*_out_ = *S*. Thus, the Hamiltonian cycle of minimum cost is *M* → *N*, *N* → *L*, *L* → *C*, *C* → *E*, *E* → *T*, *T* → *S* with *C* = 11. A generalization of the TSP, very relevant for network computing with agents, is the multiple TSP [33], which consists of determining a set of routes for *m* salesmen who all start from and turn back to a home ‘city’ (depot). Aside of the obvious relevance to traffic and scheduling, TSP is being used in applications as diverse as drilling of printed circuit boards, overhauling gas turbine engines, X-ray crystallography, computer wiring and order-picking in warehouses. A recent review [34] provides a comprehensive compendium of TSP applications.

Despite being the first NP-complete problem to be solved by brute force non-electronic computers, i.e. by DNA computing [9], and despite the Hamiltonian graph being, arguably, conceptually the closest to a physical network in its native problem form, TSP has not been solved by *physical* network computing using multiple agents, although a multicellular organism, i.e. *Physarum polycephalum*, has been used [35] to generate an approximate solution of TSP. This under-representation of solving TSP by network computing using multiple agents is even more surprising as an elaborate mathematical framework exists for the exploration of TSP networks using ant colony algorithms [36]. Instead, TSP appeared as an operational problem in running digital microfluidics, which needed to be solved by efficient algorithms [37]. The TSP has also been solved by optical networks [38], where the agents are essentially photons.

#### 2.1.5. Maze solving

*Maze-solving*, asks, given a maze (a grid of *n* × *n* regularly arranged nodes in which only connections between adjacent nodes are permitted), for a path from an entrance point to an exit point. Although the classical version is computationally tractable, i.e. requires polynomial time on a sequential computer, some versions of maze-solving, such as the simultaneous maze-solving problem [39] are NP-complete. Importantly, a great deal of experimental work has been done using many different kinds of agents to solve mazes [40–44].

Maze-solving has been classically used to experimentally assess the optimality of behavioural response, or intelligence of many organisms including ants, bees, mice, rats, octopi, and humans [45], and more recently by fungi [8,46,47], bacteria [48], *Caenorhabditis elegans* [44] and by an amoeboid [41], as well as artificial intelligence-enabled robots. Despite this very large body of experimental methodology, and very diverse use of biological agents, and despite the demonstration of the efficiency of the space searching algorithms developed by microorganisms, e.g. fungi [49], the exploration of mazes by multiple agents has not been used as a means to solve any NP-complete problem, e.g. the simultaneous maze-solving problem.

#### 2.1.6. Satisfiability problem

*The satisfiability problem* (SAT) asks, given an input Boolean formula built from variables and constraints using the NOT, AND, and OR operations, if TRUE or FALSE can consistently replace the elements of the input formula in such a way that the overall formula evaluates to TRUE. Satisfiability is an important NP-complete problem, with extremely varied applications, from ordinary ones, e.g. schedule events depending on the availability of actors and venues, and seating assignment consistent with various imposed rules, to critical decisions, e.g. design and verification of digital circuits, planning in artificial intelligence with practical use in space exploration and industrial microprocessor verification [50]. Despite this importance, and despite often using graphs to articulate relevant algorithms, SAT was not yet translated in a design of a physical network amenable to the exploration by biological agents.

*Sum-up*. There is a rather larger body of experimental work attempting to implement various solving approaches of NP-complete problems using the framework of network computing, and the majority of these efforts use a large variety of biological agents. Despite this interest, there are NP-complete problems intrinsically encoded as a network, e.g. the TSP, which have not been solved by multiple biological agents, whereas others, such as the SAT, e.g. 3-SAT, are waiting to be theoretically encoded in designs of networks amenable to the exploration by biological agents.

### 2.2. Agent run modes for network computing

**Figure 2.**
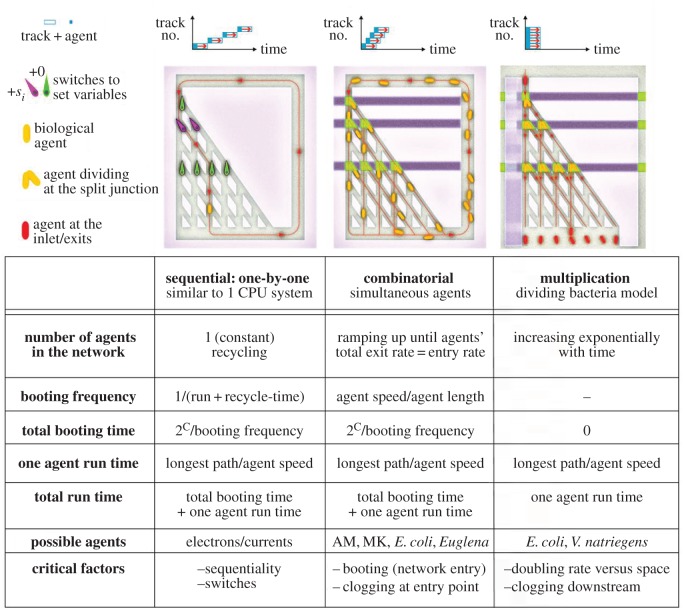
Possible operation modes in network computing. The top row shows the agents (blue squares) running in white rectangles (network tracks). The top three plots in the run mode panels show the start time delays for the various run modes. In run mode 1, the second calculation can only start after the agent finished its exploration (and, possibly, it has been recycled to the starting point). In run mode 2, the second calculation can start as soon as the first agent has left the starting point, i.e. there is physical space available for the next agent. In run mode 3, only one agent starts, and its ‘off-spring’ agents reach all the endpoints simultaneously, i.e. if all tracks would have had the same length. As the SSP network is an asymmetric triangle, the track to exit 0 is the shortest, and the track to the full sum exit is the longest. For calculating the expected computation time (see further), the latter track length (the longest arch) has been used for run time estimations.

The exploration of networks encoding combinatorial problems, such as NP-complete problems, by motile agents, can be conducted in various run modes. Figure 2 schematically presents these operational modes, taking the SSP-encoded network [19,20] as a benchmarking example. The SSP network has a triangular structure with a single starting point (top left corner in the panels in figure 2). The network features split junctions (where traffic can change direction) and pass junctions (where traffic crosses without interaction). The exits at the bottom, representing the solutions, are connected by a feedback line to the starting point (if agents are to be recycled). An agent can, therefore, be considered to be a ‘moving processor’ (a pseudo-CPU).

*The sequential run mode*. The exploration of the network by only one agent at a time is equivalent to a purely sequential processing, even if that individual agent is recycled at the end of the computation. The green and purple flippers in figure 2 represent logical switches at the split junctions, which are systematically set before every exploration run by the computing agent, in order to explore the complete parameter space. This run mode is operationally equivalent to the computing process in a typical single-core electronic computer system.

*The combinatorial run mode*. This run mode, demonstrated recently using cytoskeletal filaments [20], is essentially a concatenation of two serial processes, i.e. the feeding of the network by agents waiting in a queue, which is equivalent to the booting of the computer, and the actual computation. If the agents are fed to the computer at a frequency higher than that equivalent to the full exploration of the network by an individual agent, as in the purely sequential run mode, the computation progresses in a ‘super massively parallel’ manner, or more appropriately, in a combinatorial manner. Indeed, parallel computation processes, including ‘massively parallel’ ones, involve the processing of information by a *constant*, even if large, number of processors during the calculation, whereas for the run mode described here, the number of ‘processors’ (initially) increases as the computation progresses. Furthermore, the larger the network and the feeding frequency, the larger the number of agents (as before, the agents can also be recycled).

As an agent in the queue, before entering the network, does not need to wait for the previous one to exit the network (but just to leave the entering point), the computational network accumulates agents exploring the network in parallel. As a consequence, however, in this run mode, no switches can be operated at the split junctions, and the right- or left-direction of an agent in the split junctions is a purely stochastic process, preferably with a 50%–50% distribution, induced by a local mirror-symmetric design of the split junction. Consequently, some combinations of SSP parameters, i.e. a specific subset sum, may appear multiple times before all various combinations have been visited. In order to have a very high probability that all combinations are being explored, the number of agent runs has to be enlarged by a factor that can be estimated using the formalism of the ‘coupon collector's problem’ [51]. A major drawback of the purely combinatorial mode is that the inefficient, serial ‘upstream’ booting process will limit the overall computing time.

The total booting time can be calculated as 2*^C^* divided by the booting frequency, where the power *C* stands for the cardinality of the set, i.e. how many members the set has (as shown in figure 2). The booting frequency is derived from the agent speed divided by the (average) distance between two agents (effective body length). This distance can be chosen as the agent body length (assuming head to tail queueing), or, e.g. twice the body length of the agent (assuming a 50% duty cycle at the entering point of the network).

*The multiplication run mode*. Given the inefficient, seriality-based booting of the network computing running in combinatorial mode, a fundamentally more efficient strategy would be based on multiplication of the agents *inside* the computing network, i.e. downstream from the feeding point. Intuitively, the maximum benefit in computing time will occur if the agents multiply at every split junction. For example, bacteria could undergo cell division while exploring the network. If this would be possible, at the starting point only one computing agent would be needed, multiplying ‘on the fly’, and all the routes and exits are visited by the off-spring of the original, ‘mother’ agent. However, while the multiplication after each split junction is an ideal option, multiplication itself, at a reasonable frequency, would counter the exponential increase in the number of possible solutions versus problem size with the exponential increase in the number of computing agents. The consequences on the traffic density in the network depend on the compactness of the specific series encoded in the SSP, as will be discussed in §4.1.

## 3. Biological agents

In order to efficiently explore the networks encoding combinatorial mathematical problems, in particular NP-complete ones, the computing agents must possess several performance parameters: (i) they need to be available in large numbers, to be able to explore the whole ‘solution space’, which for problems challenging sequential computers could run in the range of millions to billions; (ii) they need to have similar dimensions, to allow standardized designs of the networks, e.g. channel widths; (iii) these dimensions are preferably small, in the nanometre or micrometre range, to allow a high density of calculations per unit area; (iv) importantly, the agents must be autonomously motile, i.e. each agent needs to possess its own propulsion, with higher speeds translating into shorter computing times; (v) the agents must not interact with each other, to enable an independent search of the ‘solution space’; (vi) while small, the agents must be independently distinguishable by a readout system; moreover, preferably the agents should be independently identifiable, i.e. each having its own ‘ID’; and (vii) they need to exhibit additional physical properties as required by the respective implementation of the computing networks, e.g. non-adherent to the walls of the microfluidic channels and non-clogging.

The computing agents asked to explore mathematically encoded networks could have an *abiotic*, or *biological* nature. In the class of abiotic agents, laminar fluids have been used [52] to ‘solve’ mazes and more recently micrometre-sized abiotic beads have been used as computing agents [18] to solve the NP-complete clique problem. However, although the beads would bring some stochasticity into the computation, they do not have independent propulsion systems, as they are carried by (and follow) the minimum pressure paths of the fluids passing through the microfluidic network, thus not exploring comprehensively (and independently) the solution space. In principle, the Janus particle technology [53], in particular self-propelling anisotropic beads [54], could fulfil many of the desiderata outlined above, but presently their application appears to be limited by their size (mm range), generation of micro-bubbles (making them ineligible for movement in microfluidic networks) and possibly shorter lifetime of movement.

In contrast to the early development of potential abiotic computing agents, *biological agents* exhibit an extremely large variety—the result of evolution in motile biological systems, from biomolecules to cells and multicellular organisms. Table 1 presents a synthetic comparison of the estimated performance of various biological systems attempting to solve SSPs.

**Table 1.** Comparison of motility parameters for various biological agents (upgraded from [55], and [19]). B/s = cell body length/s. Arch = device diagonal (mm). One agent run time (s) for SSP{2,5,9} problem in [20]. Booting frequency for double body length. Computing time (h) for SSP with cardinality 30 (first 30 prime numbers, SSP without cell division, coupon collector's correction). Bold font: high performance.

biological agents	size		velocity (µm s^−1^)	B/s (s^−1^)	arch (mm) SSP{2,5,9}	one-agent run time (s) SSP{2,5,9}	booting frequency 2BL (Hz)	computing time (h) SSPpr C30	ref.
	length (µm)	width (µm)							
cytoskeletal filaments									
actin filaments (myosin)	2	0.02	5	2.5	0.08	16	1.25	5.1 × 10^6^	[20]
microtubules (kinesin)	2	0.06	0.5	0.25	0.16	320	0.125	5.1 × 10^7^	[20]
prokaryotes									
Bacteria									
***Pseudomonas aeruginosa***	1.5	0.5	55	37	1.6	29	18.3	3.5 × 10^5^	[56]
*Chromatium okenii*	9	4.5–6	45	5	15	333	2.5	2.5 × 10^6^	[56]
***Escherichia coli***	2	0.5–1.5	16	8	1.6	100	4	1.6 × 10^6^	[56]
*Bacillus licheniformis*	3	0.8–1.3	21	7	2.6	124	3.5	1.8 × 10^6^	[56]
*Sarcina ureae*	4	2	28	7	6.4	229	3.5	1.8 × 10^6^	[57]
***Vibrio comma***	4	0.45	200	50	1.4	7	25	2.5 × 10^5^	[55]
***Vibrio natriegens***	2.5	0.4–0.6	14	5.6	1.3	93	2.8	2.2 × 10^6^	[58]
*Thiovolum majus*	15	10	600	40	32	53	20	3.2 × 10^5^	[55]
Archaea									
***Methanocaldococcus jannaschii***	1.5	0.5	380	253	1.6	4.2	127	4.9 × 10^4^	[59]
***Methanocaldococcus villosus***	1	0.5	287	287	1.6	5.6	144	4.3 × 10^4^	[59]
eukaryotes									
flagellated									
*Ceratium fusus*	420	15–30	235	0.56	48	204	0.28	2.3 × 10^7^	[60]
*Euglena viridis*	53.3	10–17	80	1.5	32	400	0.75	8.5 × 10^6^	[60]
***Monas stigmata***	6	6	270	45	19	70	22.5	2.8 × 10^5^	[60]
*Gyrodinium dorsum*	32.8	24.5	328	10	78	238	5	1.3 × 10^6^	[60]
ciliated									
*Tetrahymena* sp.	70.4	20	500	7.1	64	128	3.55	1.8 × 10^6^	[61]
*Paramecium* sp.	213	48	1000	4.7	154	154	2.35	2.7 × 10^6^	[61]
fungi									
*Neurospora crassa*	40	7	0.03	7 × 10^−4^	22	733 000	(7.5 × 10^−4^)	8.5 × 10^9^	[47]
*Pycnoporus cinnabarinus*	67	5	0.033	4 × 10^−4^	16	485 000	(4.9 × 10^−4^)	1.3 × 10^10^	[8]
nematodes									
*Caenorhabditis elegans*	1000	80	350	0.35	256	731	0.175	3.6 × 10^7^	[62]

*Cytoskeletal filaments*, which are aggregates of proteins, i.e. actin filaments, or microtubules, propelled by protein molecular motors, i.e. myosin, or kinesin, respectively, have the potential of fulfilling most of the technical requirements for motile computing agents. Indeed, both systems have been used to solve a small instance of the SSP [20]. The small size, reasonable velocity (in particular for actin filaments), distributed energy consumption (they require ATP (adenosine triphosphate) from the surrounding environment), and availability of elaborate biomolecular engineering techniques for tagging, functionalization and splitting, are among the many advantages of cytoskeletal filaments. Presently, their further use as computing agents may be hampered by the ‘open’ architecture of the microfluidic devices required for easy access and renewal of ATP, leading to computational errors due to accidental loss or addition of filaments. Finally, the technology for multiplication of filaments, required by specific designs of NP-problem-encoded networks, such as SSP, is difficult.

Because network-based computing using biological agents is a relatively new development, presently only cytoskeletal filaments have been used in proof of principle bio-computation devices. However, unlike cytoskeletal filaments with only two types of agents, *prokaryotes*, comprising the large classes of bacteria and archaea, are vastly more diverse. While usually larger than cytoskeletal filaments, some of the bacteria [55] and archaea [59] can move at very high velocities, with body lengths per second one or two orders of magnitude higher than that of cytoskeletal filaments. Although the optimum *in vitro* growth conditions are not fully known for many of these rapid swimmers, for some, e.g. *Escherichia coli*, a large body of knowledge exists, including regarding a multitude of fully described, genetically engineered mutants. Additionally, prokaryotes can live in aerobic, or anaerobic conditions, making them amenable to various growth conditions in confined spaces.

Finally, *eukaryotes* appear to have an even larger diversity than prokaryotes. However, their larger sizes, leading to larger areas required for computation, and their lower velocities relative to their body dimensions, translating into excessively long computing times, suggest that eukaryotes are unlikely to be serious contenders for efficient biological agents solving combinatorial problems. Instead, capitalizing on their more complex space searching and space partitioning strategies [8,47], eukaryotes are likely to offer insights into efficient natural algorithms, which can be subsequently reverse-engineered [49].

*Sum-up*. Network computing can benefit from an extremely large variety in biological agents of different nature, i.e. biomolecular, mono-cellular, or multicellular organisms, exhibiting various properties relevant to bio-computation, i.e. sizes, velocities, and motility mechanisms. In fact, this large variability of parameters makes the choice of biological agents for network computing difficult, as many other, less studied parameters, e.g. behaviour in confined spaces, could downgrade their expected performance in bio-computation.

## 4. Scaling of networks

### 4.1. Scaling the computing area and number of agents

The size of an SSP network is determined by (i) its unit cell size, designed for a specific computing agent; (ii) the cardinality of the problem, i.e. the number of elements in the set; and (iii) the compactness of the series, i.e. the relative distance between the numbers in the set.

The SSP unit cell size is determined by the geometrical parameters of the computing agents, e.g. width, length and secure distance between two agents. The SSP cardinality determines the number of computing agents required to solve the problem, including some additional number to offset possible errors. Consequently, for a given compactness of the series, the size and the number of computing agents needed determine the area of the SSP computing system. In principle, a larger combinatorial problem requires, by necessity, a larger number of computing agents. However, network-based computing, as described before for SSP [19,63] presents specific advantages, and disadvantages, regarding its scalability when compared to other massively parallel bio-computing approaches, e.g. DNA computing [9]. Indeed, DNA computing [9] requires an impractically large mass of DNA [13], as all the DNA mass needed for the calculation (approx. 2^C^) must be simultaneously present in the reaction step, leaving the ‘pruning’ of all combinations to a sequence of post-computation biochemical selection processes. In contrast, in network-based computation of SSP, the exploration of the 2^C^ computation paths is distributed in time and space, by recycling of agents. Consequently, network-based SSP calculation will use considerably less mass of agents, but at the expense of a much larger computation time.

Presently, network-based computing of SSP assumes [20] that the agents do not perform any function other than visiting junctions, and thus calculating various paths in the SSP-encoding network. In principle, as discussed further, the agents could perform additional functions, e.g. recording the history of their trajectories, and report on this at their exit, or in real time. However, this higher technological complexity of the agents, while valuable in accelerating the overall calculation, will not decrease the number of agents required to solve the problem, which is determined by the SSP cardinality. Moreover, it is possible that additional ‘hardware’ associated with each computing agent will increase their size, thus increasing the overall area of the computing system.

### 4.2. Complexity classes

**Figure 3.**
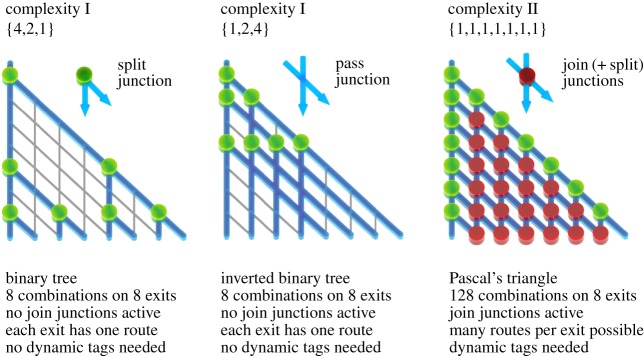
Subset sum complexity classes I and II explained in terms of split and join junctions. The complexity class I networks {4,2,1} and {1,2,4} are shown, as well as the complexity class II network {1,1,1,1,1,1,1}. (See main text in §4.2 for a detailed description.)

The SSP specifications (ii) and (iii) mentioned above determine together the total sum of the set. The compactness of the series also determines the type of complexity of the SSP network. Figure 3 presents the two complexity classes of the SSP, explained using three small example sets.

In *Complexity Class I* there is only one possible route to every legal exit, and consequently, there are only split and pass junctions active. The series in the set is strongly expanding with the cardinality. For this case, the exponential series is shown, displayed in two forms: (i) with descending numbers (binary tree) and (ii) with ascending numbers and crossing traffic lines at pass junctions, but still with the same number of routes and exits (in compliance with the commutative property of addition).

Conversely, in *Complexity Class II* there are exits that can be reached through multiple routes and, hence, there are also join junctions active. The series in the Complexity Class II sets can be very compact. For instance, the most compact series possible is Pascal's triangle. Tellingly, the set for Pascal's triangle has cardinality 7, compared to cardinality 3 for the binary tree, but occupies the same area.

The fundamental difference between the two complexity classes, i.e. single or multiple routes towards the legal exits, reveals the combinatorial (NP-complete) nature of the SSP: the solution to the problem goes beyond the discovery of the set of legal exits, also discriminating all possible routes towards these exits.

**Figure 4.**
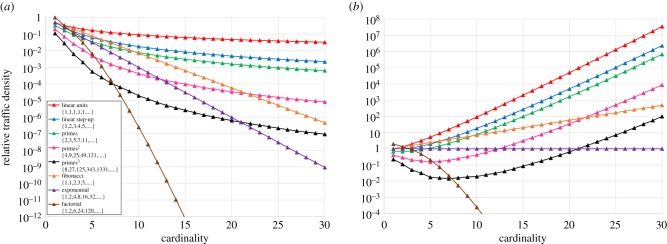
Relative average traffic densities 1/(*σ* + 1) and 2^C^/(*σ* + 1) versus cardinality *C* in subset sum networks with total sum *σ*, for respectively (*a*) the combinatorial and (*b*) the multiplication run modes, shown for the most compact series {1,1,1, …, 1} (cf. Pascal's triangle) to the Airy exponential series {1,2,4,8, …., 2^C^}, and beyond. Note that only the exponential series would show constant traffic density in case of the multiplication run mode.

The advantage of the compactness of Complexity II class comes, however, at yet another price; figure 4 presents the relative average traffic density as a function of the cardinality in series with various degrees of compactness, for the combinatorial and multiplication run modes. In the combinatorial run mode, the traffic density is falling (orders of magnitude) for all series, and the bottleneck (risk of traffic jam) is located at the starting point of the network. Conversely, in the multiplication run mode, beyond a threshold cardinality value, the traffic density is rising (again, orders of magnitude) for most series, resulting in a traffic jam further down the network. Only the exponential series would show (with multiplication at the split junctions) a constant traffic density, but at the price of an exponentially expanding network size (and consequently also an exponentially rising computation time, as will be shown in §5).

### 4.3. Scaling the readout

Solving SSP by means of network computing requires that the sequence of coordinates each and every agent passes through, or, at the very least, the sequence of the junctions it passes by, is fully recorded. This means that, until there is a reliable implementation that enables each agent to report this sequence, either ‘on the fly’, or at the end of the computation (to be addressed in §7), the overall movement of all agents needs to be tested and optically recorded, at a precision in space and time, which will not permit errors regarding the history of the positions of each agent. Consequently, the tracks of all the agents should be captured, preferably, in one optical field-of-view (FoV), and at a resolution allowing the identification of individual agents. Alternatively, if the overall computing area is too large to be visualized in one FoV, the optical recording needs to visit several sectors covering the overall movement, but at a frequency high enough to avoid confusion regarding the positioning or identity of the agents.

Three traffic scenarios should be considered, discussed in order of decreasing tracking complexity:

(i) agents can crawl over and cover each other in the channels (out-of-plane; *z*-direction);
(ii) agents can overtake each other only laterally in the channels (in the *x*–*y* plane); and
(iii) all agents move through the network channels in singular queues (no overtaking at all).

Note that channel widths and heights of less than two times the agent widths would prevent overtaking, but the risk of clogging is too large, therefore larger channel widths and heights (e.g. four times the agent widths) should be allowed in practice.

Obviously, the first scenario cannot be tracked error free, as optical tracking is performed in the *x*–*y* plane only; if one agent crawls over others, temporarily obscuring them, the tracking information becomes unreliable afterwards. Here, agents reporting their own travel history (as briefly mentioned above, and as will be elaborated in §7) would be the only way to obtain reliable traffic information; this is how—in the end—a debugged large computing system should run. The second scenario would need a pixel size smaller than the agent width (and the agent length) in order to preserve reliable traffic information when agents pass each other (e.g. on the bottom of the channel).

**Figure 5.**
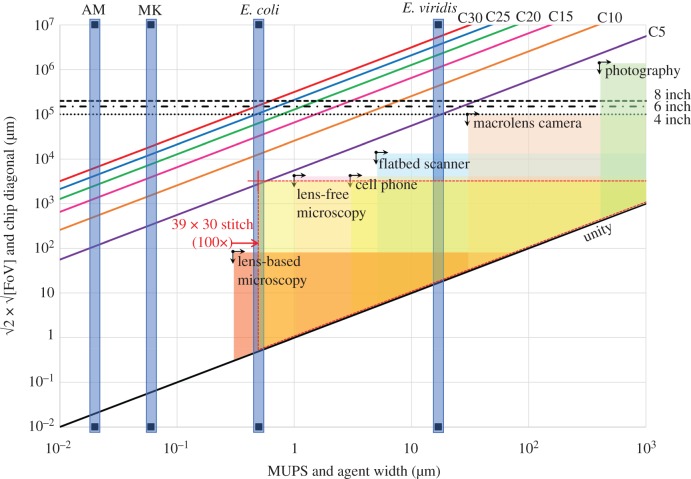
Chip size versus agent width and field of view (FoV) versus maximum useable pixel size (MUPS). The triangular work windows are shown for various microscopy techniques. (See main text in §4.3 for a detailed description.) Also shown is an example of an enlarged work window by image stitching (red cross); the limits of this method are discussed briefly further in §4.3, and in detail in the electronic supplementary material, SI-3.

In figure 5, the expected chip size is shown as a function of the agent width for the prime numbers SSP devices for various cardinalities. The horizontal black dashed lines delimit the sizes of 4-, 6- and 8-inch silicon wafers—the standards in semiconductor industry. The vertical blue bars indicate the agent width for molecular motor-driven cytoskeletal filaments, i.e. actin filaments and microtubules, as well as for small (*E. coli*) and large (*E. viridis*) microorganisms.

Because of the competition between resolution and the FoV [64], the whole imaging of the computing area requires the employment of the maximum useable pixel size (MUPS) that can still resolve individual agents, i.e. the MUPS value should be smaller than the agent width (and the agent length). The black crossed arrows indicate the intersection of the largest attainable FoV (as a square root) with the minimum attainable pixel size for various optical imaging technologies, i.e. their resolution limits. The useable optical range is obtained by the intersection of sqrt(FoV)–MUPS range with the diagonal black line indicated as ‘unity’. At the point where the top horizontal border meets the ‘unity’ line, the MUPS value is equal to the total FoV, meaning that only one pixel fits in the frame, obviously far from any reasonable application. To fully exploit the frame size available, the MUPS value should be as close as possible to the resolution limit. If the spot where the blue bars meet a specific diagonal cardinality line is inside a ‘technologically achievable’ sqrt(FoV)–MUPS triangular area, then the corresponding optical technique is, in principle, useable for monitoring the computation process using a single FoV.

It follows from figure 5 that actin and microtubule filaments are out of reach for optical monitoring, if the agent width should be resolved. The *E. coli* cell width can be resolved by a high resolution optical microscope, but the field of view would not allow more than one unit cell in one FoV. For *E. viridis,* the FoV and resolution of a flatbed scanner would allow the capture of 3 × 3 unit cells in one frame.

The third scenario is described in detail in the electronic supplementary material, SI-2, and the nomogram in electronic supplementary material, figure S1. It follows that a network with cardinality 5 for *E. viridis* can be monitored by a macro-lens equipped camera, and that the cardinality 5 network for *E. coli* and the cardinality 15 network for microtubules can be monitored by a lens-less microscope, all in one FoV, but under the naive assumption that no agents are overtaking each other in the channels.

When the area to be imaged (and monitored in time) exceeds the FoV of the imaging system, a powerful option to enlarge the effective FoV is image stitching of cyclic sampled frames. The loss of information can be minimized through faster switching speeds, which in turn are limited by the mechanical capabilities of the microscope stage. In the electronic supplementary material SI-3, the possibilities and limits of image stitching for our SSP calculation networks are modelled. In the case of high density traffic, agent speed and body length determine the sample frequency, and in the case of low density traffic, agent speed and junction distance are decisive. In electronic supplementary material, table S1, it is shown that in a typical setting used to monitor *E. coli* in the SSP prime numbers network, stitching could indeed be employed to image and monitor larger SSP networks. For traffic scenario (iii), at a resolution of 2 µm with a 10× objective, instead of a cardinality 4 network in one FoV, a cardinality 15 network can be monitored in time by (cyclic) stitching of 14 × 11 frames (shown by a red cross in electronic supplementary material, figure S1). For traffic scenario (ii), at a resolution of 0.5 µm with a 100× objective, a cardinality 5 network can be monitored in time by (cyclic) stitching of 39 × 30 frames (shown by a red cross in figure 5).

One corollary of the above analysis is that, for *E. coli*, or an agent with similar motility and size parameters, the area of a device solving a prime numbers SSP with a cardinality of 30 is slightly larger than a 6 inch wafer. As this area cannot be captured in one FoV by any known optical monitoring system with the proper resolution needed, the readout would need an array of 60 × 60 frames of a lens-less microscope being continuously switched in order to keep track of all agents simultaneously. Even if that were technically feasible, the data storage needed would be very large. Moreover, from a fabrication point of view, such large chips are very vulnerable to fatal errors by dust particles in the lithographic steps. Indeed, one dust particle on a wafer with 100 chips lowers the yield from 100 to 99%, but on a one-chip wafer it leads to 0% yield (i.e. 100% failure). Alternatively, a purposefully designed and fabricated optical chip, built in the ‘floor’ of the computing chip, with pixels smaller than half the channel width (or even better, half the agent width), and covering only the actual computing area, is a technologically achievable, albeit non-trivial solution.

*Sum-up*. It appears that the scaling of networks, in particular for solving SSP, is the most problematic, albeit technological and not fundamental, aspect of network computing with biological agents. Indeed, the chip area, which grows with the size of the problem, requires FoVs which are not presently available. Alternatively, to limit the explosion of the chip area with the size of the problem would require smaller agents, which in turn would require a higher resolution, but this would further raise problems for the achievable FoV. Ultimately, a technology that allows the agents to report their own travel history (at the exits) would not need optical recording of the total network.

## 5. Computing time

### 5.1. Computing time versus run modes

**Figure 6.**
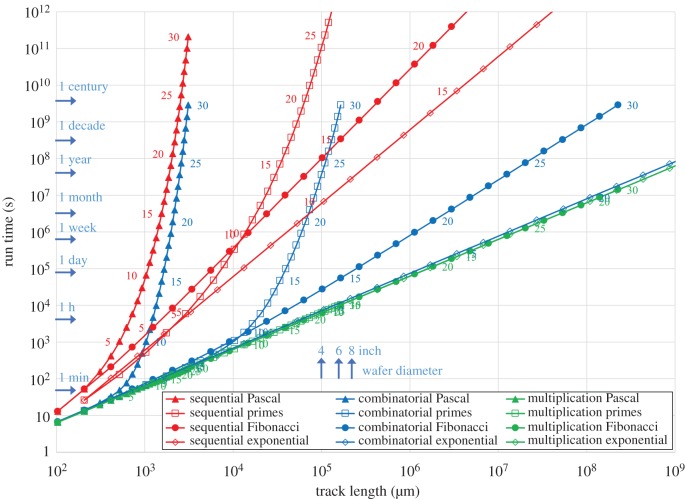
Run time versus track length as a function of cardinality for subset sum networks with 2 µm track width, 16 µm s^−1^ agent speed and 2 µm body length (*E. coli*). A correction factor derived from the ‘coupon collector's problem’ [51] (§2.2) was included to deal with the stochastic nature of the parameter exploration in the combinatorial run mode.

In the first instance, the time to solve an SSP depends on the mode of operation of the computing agents, the extent of the series, i.e. its cardinality, and the structure of the series of numbers. More compact series will result in a smaller computing area and consequently a shorter computing time. Figure 6 presents the relationship between the estimated computing times for *E. coli* (table 1) in the three run modes (detailed in §2.2) versus the longest track in the SSP chips for four number series (four compactness types: Pascal series, prime numbers, Fibonacci series and exponential numbers) and cardinality (shown as a label in steps of 5 next to the calculated points). For a given series and given cardinality, the track length is the same for all run modes.

As expected, the highest computing times are observed for the sequential run mode, and the lowest are observed for the multiplication run mode. The difference in run time between the sequential and the combinatorial run modes is small for compact series, but quite large for expanding series. Importantly, in the combinatorial run mode, the estimated run times at higher cardinality become independent of the compactness of the series, due to the fact that the total booting time needed to accommodate large numbers of agents in the network is orders of magnitude larger than the time needed to run a single track (compare also the booting frequency data in table 1, explained in figure 2, §2.2). The multiplication run modes for the various series are all following the same straight line because, effectively, only one agent starts and the off-spring that takes the longest track is monitored, but all are assumed to run with the same average speed.

In figure 6 the sizes of 4, 6 and 8 inch wafers are indicated (by blue arrows) for the possible fabrication limits. A network with cardinality 30 would only fit on a standard wafer for the prime number (and the Pascal) series. Also indicated, by blue arrows, are time frames. Only the multiplication run mode would allow a cardinality 30 network to be run in a reasonable time.

### 5.2. Benchmarking biological agents based network computing with electronic computing

While electronic computers perform computations in a serial manner, they are many orders of magnitude faster per operation than it is reasonable to expect from network computing with biological agents. Consequently, the immediate scaling question is to what extent an *ideal* set of agent parameters, i.e. size, speed, multiplication rate, which inform the design of the computing network, would make network computing using biological agents possibly competitive with the electronic computers. In order to have a comparison between the ideal performance of network computing with biological agents, and electronic computers, two sets of simulations have been performed.

At this junction an important distinction must be made when comparing the performance of electronic computers with any other alternative computing devices, including the one recently proposed for solving SSP [20]. It was argued [65] that SSP has a known solution that runs in O(NT) time, and that there are algorithms, e.g. Pisinger's [66], which can solve SSP very quickly if run by electronic computers. However, the alternative computation approaches, including DNA, quantum, and networks-based computing, to name a few, propose in the first instance computing *devices* with associated *operational procedures*, rather new algorithms, which indeed might be required to be developed to capitalize on the potential benefits offered by the new computing hardware. Consequently, and taking into consideration the tentative or early stage of development of the new computing devices, any meaningful comparison of the computing power of electronic computers and any new paradigmatic computing device must use comparative algorithmic procedures, rather than the most advanced ones, which by virtue of decades long history of microelectronics have been solely and specifically created and optimized for sequential electronic computers.

On this background, a computer program was designed to solve the SSP by brute force (i.e. no efficient ’heuristic’ algorithms have been used) for electronic computers, using emulators of various generations of computer chips. To ensure a more conservative approach, the program has been coded in C++ to allow the maximum use of computer chip RAM, low-level memory access, efficient mapping to machine instructions and flexibility. The program is described in detail in the electronic supplementary material, SI-4. Separately, the operation of a network computer using biological agents, both used before and hypothetical, has been simulated for selected agents from table 1, for cardinalities considerably larger than presently possible in experiments. The program is described in detail in the electronic supplementary material, SI-5.

**Figure 7.**
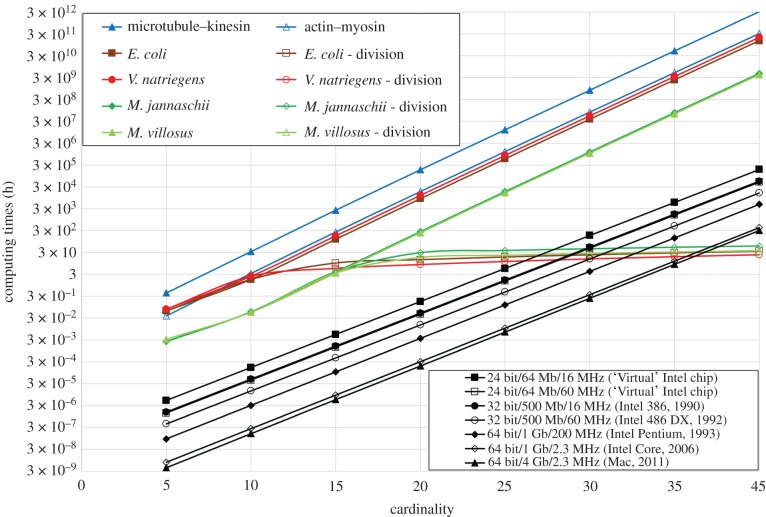
Comparison of the computing performance of the electronic computers (bottom-right half) and biological computers (top-left half) solving the prime numbers subset problem. Correction factors for the ‘coupon collector's problem’ and for a 45–55% instead of 50–50% split junction distribution were included.

Figure 7 presents the estimated run times for solving an SSP by means of network computing [20], with various cardinalities, using (i) biological agents, either cytoskeletal filaments, i.e. actin filaments propelled by myosin and microtubules propelled by kinesin, or hypothetically, several bacterial agents (exhibiting superior parameters): *M. jannaschii*, which has a high speed, *M. villosus*, which, due to its small size, calculates faster, *V. natriegens*, which multiplies frequently and—as a reference—*E. coli* (table 1), and (ii) various generations of computer chips i.e. Intel's 286, 386, 486 and single core Pentium and a present-day MacBook chip. As opposed to all electronic chips, which perform computation in a sequential run mode, the simulated computation by biological agents is performed in the combinatorial run mode, for cytoskeletal filaments and the chosen bacterial agents, and in the multiplication run mode for the latter, assuming multiplication rates reported in the literature; the following doubling times have been used: *M. jannaschii*: 74 min, *M. villosus*: 45 min, *E. coli*: 30 min and *V. natriegens*: 15 min.

Even a cursory inspection of the computing performance comparison of the electronic and network-based computers reveals several evident trends.

— As a group, the electronic chips, operating in the sequential run mode, outperform—by a few orders of magnitude—the biological ones operating in a combinatorial run mode. Moreover, this performance gap remains constant, or increases slightly, throughout the range of cardinalities tested. Indeed, even if electronic computers operate in the sequential run mode (figure 2), they also operate at clock frequencies of the order of GHz, whereas the bio-computers will operate at typically 0.1–10 Hz (table 1).
— The difference in performance of electronic computers computing SSP, i.e. in [20] which used RAM-intensive software (MATLAB), versus the present study, which inheres from a more efficient use of RAM, also reveals the importance of the allocation of chip memory, a perennial problem for electronic devices. It is important to observe that eventually any electronic chip solving SSP (or any NP-complete problem) will hit an intrinsic ‘memory wall’ [67] when all chip memory is used for ever larger problems. Note that for the cardinality at which this happens, it will result in a truncation of the black lines in figure 7 at the right-hand side. In contrast, and aside from other physical limitations (assessed in the previous sections, e.g. chip area, readout), network computing using biological agents should not experience any similar ‘memory wall’.
— The motility speed of the biological agents appears to have only a secondary, albeit positive, effect on the performance of network computing, but by itself it will not be able to make the bio-computer outperform the electronic one. Indeed, the speed of the faster biological agent, i.e. *M. villosus*, would need to be raised from its already high value of 287 µm s^−1^ three orders of magnitude, i.e. approximately 30 cm s^−1^, only to catch up with the slowest electronic chip tested, i.e. Intel's 286. On the other hand—and crucially—it is estimated that bio-computers operating in the multiplication mode quickly outperform electronic chips, almost independent of the clock speed of the latter, as shown in figure 7.

*Sum-up*. While some improvement can be achieved, in principle, using faster biological agents operating in the pure combinatorial run mode, the computing performance of electronic computers will remain unmatched for the foreseeable future. While more analysis would be required to explore the possible collapse of performance of (single core) electronic computers for larger SSPs (‘memory wall’), a more advantageous avenue will likely be based on the use of biological agents running in a multiplication run mode.

## 6. Scaling the energy required for computation

With alternatives to ‘classical’ electronic computers not being fully demonstrated, or most likely being in early stages of development, presently only high performance computation (HPC) is the closest to tackling large scale combinatorial problems. However, in itself, the scale of combinatorial or complex problems of practical importance translates into large amounts of energy used, if the computation is performed by sequential electronic computers. For instance, solving large complex problems, even if not necessarily combinatorial in nature, would require scaling up HPC to exascale computing, i.e. 10^18^ floating-point operations per second (Flops) [68]. However, as the most powerful supercomputer, Sunway TaihuLight, requires 42 MW of power (an average hydroelectric facility generates 57 MW), scaling it to exascale regime would require 450 MW, with running costs of US$270 million per year [69]. Arguably, a similar result regarding energy consumption would be obtained for a technical solution involving myriads of smaller scale PCs interconnected in a very large computer network, such as a very large ‘computer farm’. Consequently, and aside from the difficulty of solving large combinatorial problems, it appears that electronic computers are also unsustainable energy-wise.

The computational systems able to solve, in principle, combinatorial problems can be aggregated into three classes (table 2). The most energy-efficient systems are, expectedly, *molecular computers*, of which the most well-known is DNA computing [9], followed by numerous variations [74]. Indeed, since in molecular computers the mathematical operations *are*, actually, chemical reactions, the energy/operation required by DNA computing is the closest to the thermodynamic limit calculated elsewhere [72]. At the opposite end of the spectrum considered, *silicon-based computers*, including ‘classical’ HPC and quantum computing, are seven to eleven orders of magnitude more energy consuming, per operation, compared to molecular computing (and there are two orders of magnitude between the most performant HPC system and an advanced quantum computing system).

**Table 2.** Energy efficiency of various computing systems. Notes. (1) Top computing speed for top 500 supercomputers in 2017. (2) Top energy efficiency for top 500 supercomputers in 2017. (3) Pumping energy for a chip with *d* = 200 nm × *L* = 1000 nm (cf. [20]) using capillarity principles will result in considerably lower energy consumption. (4) Energy consumption estimated using the general formula for energy consumption in motility of prokaryotes [70,71].

system	implementation	measurement	energy J/operation	explanations, [sources], (notes)
molecular computers	thermodynamic limit	theory	2.90 × 10^−22^	thermodynamics [72]
	DNA	estimated	5.00 × 10^−20^	thermodynamics [9]
Si-based computing	electronic	actual	1.65 × 10^−10^	Sunway TaihuLight [73], (1)
		actual	5.88 × 10^−11^	Shoubu system B [73], (2)
	quantum	estimated	2.00 × 10^−13^	DWave system [69]
computing with networks	microfluidics	estimated	1.29 × 10^−12^	beads in microfluidics [18], (3)
	cytoskeletal filaments/molecular motors	estimated	4.95 × 10^−14^	kinesin/microtubules [20]
		estimated	2.00 × 10^−14^	myosin/actin filaments [20]
	microorganisms	estimated	1.43 × 10^−13^	*Escherichia coli* (4)
		estimated	2.76 × 10^−13^	*Vibrio natriegens* (4)
		estimated	8.67 × 10^−14^	*Methanocaldococcus jannaschii* (4)
		estimated	1.16 × 10^−13^	*Methanocaldococcus villosus* (4)
		estimated	2.01 × 10^−9^	*Euglena viridis* (4)

Importantly, the energy consumption for silicon-based computers reported in table 2 includes only the energy required for ‘core phase’, that is, for the section of the workload that undergoes parallel execution. It typically does not include the parallel job launch and teardown, which is required to run for at least one minute. Consequently, no energy consumption is reported for environmental, e.g. cooling, and auxiliary, e.g. lighting, needs. Finally, systems performing *computing with agents exploring networks* present an estimated energy consumption/operation in between molecular and Si-based computing, but over a very large range, i.e. between thirteen and six orders of magnitude higher than molecular computers. As with silicon-based computers no energy consumption is estimated other than that for computation proper. Within this class is microfluidics-based computation, which relies on beads being pushed, with some level of randomness, through networks encoding an NP-complete problem [18] (although it is possible that the energy required by microfluidics-based computation could be decreased substantially by using capillary-driven flows). The exploration of a network by larger microorganisms, e.g. *Euglena*, has an energy consumption/operation similar to the Si-based computers. However, the use of nano- or small micrometre-sized biological agents, i.e. cytoskeletal filaments [20], or bacteria [70,71], respectively, is estimated to bring the energy consumption/operation one order of magnitude down, or similar to that of an advanced, and energy consumption-competitive quantum computing system.

*Sum-up*. The estimated energy consumption per operation for network-based computing using micro- or nanometre sized biological agents is in the range of 10^−14^–10^−13^ J/operation (and much larger for tens of micrometre-sized agents), which is similar to the reported energy performance of quantum computation, and three to four orders of magnitude better than present supercomputers. Additionally, biological computers would have the advantage of distributed energy consumption, in contrast with Si-based computers, including quantum computers.

## 7. Perspectives and future work

The scaling analysis presented above identifies several challenges ahead for the further development of network computing using biological agents, taking the solving of SSP as a benchmark case. These challenges are either fundamental or related to the underdevelopment of the presently available service technologies required. To this end, further areas of research and development, as well as under-used opportunities, are as follows.

Recording the traffic history on each individual agent

Unlike SSPs of Complexity Class I (figure 3), Complexity Class II problems constitute ‘true’ combinatorial problems. The consequence is that agents that have taken different routes towards the same exit have to be clearly discriminated in order to be able to solve the combinatorial problem. To demonstrate, by counter-example, the SSP presented in figure 2 can be replaced by a very fast electronic device, consisting of parallel arrays of switching transistors (electronic supplementary material, SI-6). This device, which has all the architectural characteristics of a network-based computer, but which lacks the capability to differentiate between computing agents, shows the correct exits essentially instantaneously, but as the ‘agents’, i.e. the electrons, are ‘anonymous’, the routes of the individual agents cannot be discriminated. Consequently, this very fast device is not truly able to solve combinatorial problems. The area and the energy needs of this device scale quadratically with the total sum in unary coded form, which in turn scales exponentially with the regular binary coded representation of numbers used in sequential electronic computers.

Individual bacterial agents in a network can be monitored by video tracking techniques, but for higher cardinality problems, the amount of (image) data to be stored and interpreted will rise exponentially. For instance, for a cardinality 30 problem, more than 1 billion agents will have to be tracked simultaneously.

Instead of the troublesome high resolution tracking (in time) of identical agents simultaneously, as described in §4.3, one could discriminate each individual agent by adding a unique static label (i.e. a label that is not changed during the run time of the experiment) and lower down the image capturing frequency. However, still full (video) tracking of all the agents would be necessary to retrieve all the routes taken by individual agents. For instance, for a cardinality 30 problem more than 1 billion agents with unique labels would be needed—a clear technological impossibility. As there are most probably not enough unique labels available, one could try to employ a limited selection of labels to compose binary coded ‘words’ (2^30^ words by using 30 labels). For very large networks, however, the process of coding and decoding of the labels may constitute in itself an operation rising exponentially in time.

**Figure 8.**
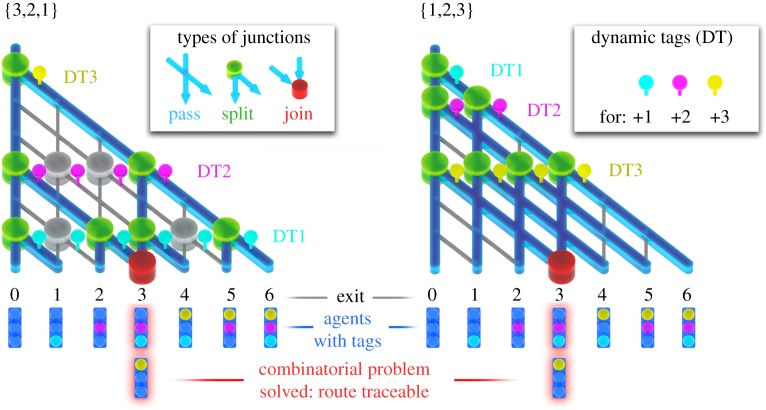
Dynamic tags for solving combinatorial problems in the very small complexity class II networks {1,2,3} and {3,2,1} are shown. (See main text in §7 for a detailed description.)

A dynamic labelling system ‘Tag & Trace’, however, could store the necessary information about the route followed by the individual agent, on the agent itself, as shown in figure 8. At every split junction the agent proceeding in the direction associated with the addition of that particular number will get a label (a ‘stamp’, or a tag). At the exits, the agents will be interrogated as to which labels have been collected on the route towards that particular exit. In this way, the routes of individual agents arriving at joined exits can be discriminated, and combinatorial problems can be solved. Although for a cardinality 30 problem still more than 1 billion agents with 1 billion label ‘words’ composed of 30 unique labels collected ‘on the fly’ would be needed, the video tracking (with the image data explosion and image stitching, described in §4.2) would no longer be necessary.

Importantly, when multiplication of agents in the network can be employed to address all possible combinations (in linear time), as e.g. in the proposed multiplication run mode, it is essential that all labels collected ‘on the fly’ are precisely copied at every multiplication event, or otherwise the information about the route—taken so far—is lost.

If the traffic density rises in compact networks, as shown in figure 4, and therefore clogging may occur, other methods for obtaining massively parallel operations need to be considered. When the multiplication of the agents cannot be employed to address all possible combinations, the total calculation time can be lowered by parallel processing.

**Figure 9.**
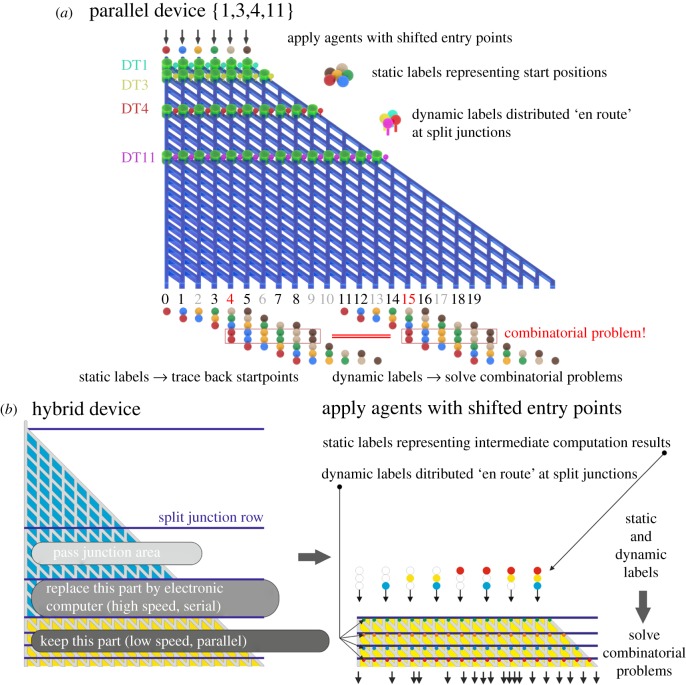
Static tags for parallel computation in: (*a*) a parallel device and (*b*) a hybrid bio-electronic device. Additionally, dynamic tags are needed for solving combinatorial problems. (See main text in §7 for a detailed description.)

In the combinatorial run mode, the choice of the combinations explored by the agents is a stochastic process. Therefore, the total calculation can be performed in a shorter time using distribution over multiple identical networks (parallel processing). This can be done on separate chips, or by applying parallel traffic in one network. In figure 9*a* a parallel subset sum computation device is shown, where the agents are applied to various shifted starting points. At every starting point, a unique static label is attached to the agents. After running through the network, at every exit the labels are checked, and in this way the starting point can be retrieved, and the effective exit number obtained. Apart from the static labelling method for enabling parallel processing, dynamic labelling is needed simultaneously for solving combinatorial problems. After running through the network, at every exit the static and dynamic labels present on each and every agent have to be checked ‘on the fly’.

Additionally, when multiplication of the agents cannot be employed to address all possible combinations, another option to speed up solving combinatorial problems consists of employing the best of two worlds [63]: in figure 9*b* a subset sum network is displayed with descending numbers. A hybrid device could be created by replacing the first part of the network by an electronic computer (serial, but high speed) while leaving the rest of the device for the bio-computer (low speed, but parallel). The agents are applied with shifted entry points calculated from the intermediate computation results of the electronic computer, and static labels are applied to the agents, in which labels code for the virtual ‘route’ taken so far, as calculated by the electronic computer. Apart from this static labelling, the dynamic labelling is again needed in the bio-computer part of the network for solving combinatorial problems. Likewise, after running the biological part of the network, at every exit the static and dynamic labels present on each and every agent must be checked ‘on the fly’.

*Development of new designs of computing networks*:

— While the proposed network computing approach for solving SSP [20] uses a brute force method implemented through a *physical* device, it has been argued [65] that SSP can be efficiently solved by efficient software *algorithms* without the need for alternative elaborate hardware. Consequently, SSP is likely to remain a benchmark method testing the prowess of various combinatorial computation methods [63], rather than finding other, more tangible applications. This limitation demonstrates the need for further approaches for encoding of other NP-complete problems in graphs, and subsequently into networks and computation devices, with immediate, but not exclusive, examples being the TSP and 3-SAT.
—  The fundamentally new designs will also benefit from further, second order improvements, e.g. better area compactness using a three-dimensional architecture of the chip, as proposed for cytoskeletal filaments [75], dynamic logical gates, as opposed to the present static pass and split junctions [20], and hybrid electronic/network-based devices [20].

*Biological agents and operation modes*:

—  The immediate realization when contemplating the parameters of biological agents and their possible run modes is that, at least for solving SSP via the proposed approach [20], the only pathway to achieve a better computing speed than electronic computers is to enable biological agents run in the multiplication mode. Indeed, as the chips encoding NP-complete problems grow exponentially in some parameter, e.g. area or number of agents, the only option to counterbalance this trend is to use another exponential, i.e. multiplication. The multiplication of biological agents occurs naturally for microorganisms, but could be achieved, in principle, with cytoskeletal filaments too, by hijacking the biomolecular treadmilling.
—  Separately, it should be noted that solving SSP by network computing using biological agents in the combinatorial run mode does not suffer from the scaling limitations regarding the mass of agents, which is the major bottleneck in DNA computing [13], where all the DNA mass needed for the calculation must be simultaneously present in the reaction step (§4.1). In networks-based computation of SSP, the exploration of the computations paths is distributed in time and space by recycling of agents. Consequently, network-based SSP calculation will use considerably less mass of agents, but at the expense of a much larger computation time.
—  The strategy of hard-wiring of computing tasks into a physical device should be extended to the computing agents. Indeed, presently the agents are passively exploring the allowable paths, translating into difficult to achieve tasks for the readout system, but in principle biological agents, if appropriately tagged, e.g. using fluorophores responsive to the local environment, can perform computing tasks ‘on the fly’, e.g. by recording autonomously the history of the visited gates (figure 8), or by ‘beaming up’ events, as previously proposed for cytoskeletal filaments [76,77].

*Tug of war between area and readout*:

—  The only option for solving large SSPs is to design and fabricate wafer-large optical chips, which is in principle achievable with present technology, but at a high cost and with high fabrication failure risk. A possible improvement would be to provide the readout, at the appropriate resolution, in the network paths only.
—  An alternative would be to switch from an area-based, to an agent-based readout, if as suggested above, the biological agents might record their travelling history. This readout option is indeed used by DNA computing, with the difference that in the network computing case only a smaller number of agents would be interrogated at a time. Also, it is very likely that the optical readout, which is fast, would remain the technology of choice.

*Energy*:

—  The estimated energy consumption per operation is already competitive with electronic computers, but there are various opportunities to increase this energetic efficiency. For instance, actual measurements of energy consumption, instead of the estimations (table 2) can reveal better energetic efficiency, in particular for biological agents belonging to the Archaea.
—  Another energy-related area is the *sustainability* of computation, rather than its energetic efficiency. Indeed, while *E. viridis* appears to use orders of magnitude more energy than other biological agents (table 2), it can use light as a source of energy [78].

## 8. Conclusion

In brute force computing, for a problem of input size *C*, 2^C^ solutions have to be addressed, and this workload needs to be distributed in space and time: something has to give. The technological challenges related to scaling up the size of the problems have been identified in terms of chip fabrication, readout reliability and energy efficiency. The necessary computing time of parallel operating biological agents has been compared to the electronic single CPU computers. Labelling of biological agents ‘on the fly’ with accompanying readout of their travel history at the exit seems a promising new development avenue for tackling combinatorial problems.

## Supplementary Material

Supplementary Information

## Data accessibility

Additional data can be found in the electronic supplementary material.

## References

[1] Nam GJ, Sakallah KA, Rutenbar RA 2002 A new FPGA detailed routing approach via search-based Boolean satisfiability. IEEE Trans. Comput. Aided Des. Integr. Circuits Syst. 21, 674–684. (10.1109/TCAD.2002.1004311)

[2] Fraenkel AS 1993 Complexity of protein folding. Bull. Math. Biol. 55, 1199–1210. (10.1007/BF02460704) 8281132

[3] Pierce NA, Winfree E 2003 Protein design is NP-hard. Protein Eng. 15, 779–782. (10.1093/protein/15.10.779) 12468711

[4] Hopfield JJ, Tank DW 1985 ‘Neural’ computation of decisions in optimization problems. Biol. Cybern. 52, 141–152. 4027280 10.1007/BF00339943

[5] Massacci F 1996 Contextual reasoning is NP-complete. In Proc. 13th National Conf. on Artificial Intelligence, Portland, OR, USA, 4–8 August 1996, vol. 1, pp. 621–626.

[6] Brandes U, Delling D, Gaertler M, Gorke R, Hoefer M, Nikoloski Z, Wagner D 2008 On modularity clustering. IEEE Trans. Knowl. Data Eng. 20, 172–188. (10.1109/TKDE.2007.190689)

[7] Aaronson S 2005 Guest Column: NP-complete problems and physical reality. ACM SIGACT News 36, 30–52. (10.1145/1052796.1052804)

[8] Hanson KL, Nicolau DV, Filipponi L, Wang L, Lee AP, Nicolau DV 2006 Fungi use efficient algorithms for the exploration of microfluidic networks. Small 2, 1212–1220. (10.1002/smll.200600105) 17193591

[9] Adleman LM 1994 Molecular computation of solutions to combinatorial problems. Science 266, 1021–1024. (10.1126/science.7973651) 7973651

[10] Lipton R 1995 DNA solution of hard computational problems. Science 268, 542–545. (10.1126/science.7725098) 7725098

[11] Mao C, Labean TH, Reif JH, Seeman NC 2000 Logical computation using algorithmic self-assembly of DNA triple-crossover molecules. Nature 407, 493–496. (10.1038/35035038) 11028996

[12] Qian L, Winfree E 2011 Scaling up digital circuit computation with DNA strand displacement cascades. Science 332, 1196–1201. (10.1126/science.1200520) 21636773

[13] Beaver D 1995 Computing with DNA. J. Comput. Biol. 2, 1–7. (10.1089/cmb.1995.2.1) 7497113

[14] Braich RS, Chelyapov N, Johnson C, Rothemund PWK, Adleman L 2002 Solution of a 20-variable 3-SAT problem on a DNA computer. Science 296, 499–502. (10.1126/science.1069528) 11896237

[15] Ouyang Q, Kaplan PD, Liu S, Libchaber A 1997 DNA solution of the maximal clique problem. Science 278, 446–449. (10.1126/science.278.5337.446) 9334300

[16] Reif JH 2011 Scaling up DNA computation. Science 332, 1156–1157. (10.1126/science.1208068) 21636761

[17] Ladd TD, Jelezko F, Laflamme R, Nakamura Y, Monroe C, O'brien JL 2010 Quantum computers. Nature 464, 45–53. (10.1038/nature08812) 20203602

[18] Chiu DT, Pezzoli E, Wu H, Stroock AD, Whitesides GM 2001 Using three-dimensional microfluidic networks for solving computationally hard problems. Proc. Natl Acad. Sci. USA 98, 2961–2966. (10.1073/pnas.061014198) 11248014 PMC30589

[19] Nicolau DV, Nicolau DV, Solana G, Hanson KL, Filipponi L, Wang L, Lee AP 2006 Molecular motors-based micro- and nano-biocomputation devices. Microelectron. Eng. 83, 1582–1588. (10.1016/j.mee.2006.01.198)

[20] Nicolau DV et al. 2016 Parallel computation with molecular-motor-propelled agents in nanofabricated networks. Proc. Natl Acad. Sci. USA 113, 2591–2596. (10.1073/pnas.1510825113) 26903637 PMC4791004

[21] Caulfield HJ, Dolev S 2010 Why future supercomputing requires optics. Nat. Photonics 4, 261–263. (10.1038/nphoton.2010.94)

[22] Schnorr CP, Euchner M 1994 Lattice basis reduction: improved practical algorithms and solving subset sum problems. Math. Program. 66, 181–199. (10.1007/BF01581144)

[23] Kate A, Goldberg I 2011 Generalizing cryptosystems based on the subset sum problem. Int. J. Inf. Secur. 10, 189–199. (10.1007/s10207-011-0129-2)

[24] Badawi AA, Veeravalli B, Aung KMM, Hamadicharef B 2018 Accelerating subset sum and lattice based public-key cryptosystems with multi-core CPUs and GPUs. J. Parallel Distrib. Comput. 119, 179–190. (10.1016/j.jpdc.2018.04.014)

[25] Fréville A 2004 The multidimensional 0–1 knapsack problem: an overview. Eur. J. Oper. Res. 155, 1–21. (10.1016/S0377-2217(03)00274-1)

[26] Darmann A, Nicosia G, Pferschy U, Schauer J 2014 The Subset Sum game. Eur. J. Oper. Res. 233, 539–549. (10.1016/j.ejor.2013.08.047) 25844012 PMC4375680

[27] Oltean M, Groşan C, Oltean M 2004 Designing digital circuits for the knapsack problem. In *Computational science: ICCS 2004* (eds M Bubak, GD van Albada, PMA Sloot, J Dongarra). Lecture Notes in Computer Science, vol. 3038, pp. 1257–1264. Berlin, Germany: Springer (10.1007/978-3-540-24688-6_162)

[28] Oltean M, Muntean O 2009 Solving the subset-sum problem with a light-based device. Nat. Comput. 8, 321–331. (10.1007/s11047-007-9059-3)

[29] Wu Q, Hao JK 2015 A review on algorithms for maximum clique problems. Eur. J. Oper. Res. 242, 693–709. (10.1016/j.ejor.2014.09.064)

[30] Hwang FK, Richards DS 1992 Steiner tree problems. Networks 22, 55–89. (10.1002/net.3230220105)

[31] Liu L, Song Y, Zhang H, Ma H, Vasilakos AV 2015 Physarum optimization: a biology-inspired algorithm for the Steiner tree problem in networks. IEEE Trans. Comput. 64, 819–832.

[32] Nakagaki T, Kobayashi R, Nishiura Y, Ueda T 2004 Obtaining multiple separate food sources: behavioural intelligence in the Physarum plasmodium. Proc. R. Soc. Lond. B 271, 2305–2310. (10.1098/rspb.2004.2856) PMC1691859 15539357

[33] Bektas T 2006 The multiple traveling salesman problem: an overview of formulations and solution procedures. Omega 34, 209–219. (10.1016/j.omega.2004.10.004)

[34] Matai R, Singh S, Mittal ML 2010 Traveling salesman problem: an overview of applications, formulations, and solution approaches. In Traveling salesman problem (ed. Davendra D). IntechOpen (10.5772/12909)

[35] Jones J, Adamatzky A 2014 Computation of the travelling salesman problem by a shrinking blob. Nat. Comput. 13, 1–16. (10.1007/s11047-013-9401-x)

[36] Dorigo M, Gambardella LM 1997 Ant colony system: a cooperative learning approach to the traveling salesman problem. IEEE Trans. Evol. Comput. 1, 53–66. (10.1109/4235.585892)

[37] Mitra D, Roy S, Bhattacharjee S, Chakrabarty K, Bhattacharya BB 2014 On-chip sample preparation for multiple targets using digital microfluidics. IEEE Trans. Comput. Aided Des. Integr. Circuits Syst. 33, 1131–1144. (10.1109/TCAD.2014.2323200)

[38] Wu K, De Abajo JG, Soci C, Shum PP, Zheludev NI 2014 An optical fiber network oracle for NP-complete problems. Light Sci. Appl. 3, e147.

[39] Funke S, Nusser A, Storandt S 2017 The simultaneous maze solving problem In 31st AAAI Conference on Artificial Intelligence, AAAI 2017.

[40] Held M, Edwards C, Nicolau DV 2008 Examining the behaviour of fungal cells in microconfined mazelike structures Proc. SPIE 6859, 68590U (10.1117/12.759453)

[41] Nakagaki T, Yamada H, Tóth Á 2000 Maze-solving by an amoeboid organism. Nature 407, 470 (10.1038/35035159) 11028990

[42] Ntinas V, Vourkas I, Sirakoulis GC, Adamatzky AI 2017 Oscillation-based slime mould electronic circuit model for maze-solving computations. IEEE Trans. Circuits Syst. Regul. Pap. 64, 1552–1563. (10.1109/TCSI.2016.2566278)

[43] Paolillo A, Faragasso A, Oriolo G, Vendittelli M 2017 Vision-based maze navigation for humanoid robots. Auton. Robots 41, 293–309. (10.1007/s10514-015-9533-1)

[44] Qin J, Wheeler AR 2007 Maze exploration and learning in C. elegans. Lab. Chip 7, 186–192. (10.1039/B613414A) 17268620

[45] Jellinger KA 2007 Comparative cognition: experimental exploration of animal intelligence. Eur. J. Neurol. 14, e34 (10.1111/j.1468-1331.2007.01840.x)17222090

[46] Held M, Binz M, Edwards C, Nicolau DV 2009 Dynamic behaviour of fungi in microfluidics—a comparative study Proc. SPIE 7182, 718213 (10.1117/12.822464)

[47] Held M, Edwards C, Nicolau DV 2011 Probing the growth dynamics of Neurospora crassa with microfluidic structures. Fungal Biol. 115, 493–505. (10.1016/j.funbio.2011.02.003) 21640314

[48] Park S, Wolanin PM, Yuzbashyan EA, Silberzan P, Stock JB, Austin RH 2003 Motion to form a quorum. Science 301, 188 (10.1126/science.1079805) 12855801

[49] Asenova E, Lin HY, Fu E, Nicolau DV, Nicolau DV 2016 Optimal fungal space searching algorithms. IEEE Trans. Nanobioscience 15, 613–618. 27187968 10.1109/TNB.2016.2567098

[50] Malik S, Zhang L 2009 Boolean satisfiability from theoretical hardness to practical success. Commun. ACM 52, 76–82. (10.1145/1536616.1536637)

[51] Boneh A, Hofri M 1997 The coupon-collector problem revisited—a survey of engineering problems and computational methods. Commun. Stat. Part C Stoch. Models 13, 39–66. (10.1080/15326349708807412)

[52] Fuerstman MJ, Deschatelets P, Kane R, Schwartz A, Kenis PJA, Deutch JM, Whitesides GM, 2003 Solving mazes using microfluidic networks. Langmuir 19, 4714–4722. (10.1021/la030054x)

[53] Walther A, Müller AHE 2013 Janus particles: synthesis, self-assembly, physical properties, and applications. Chem. Rev. 113, 5194–5261. (10.1021/cr300089t) 23557169

[54] Howse JR, Jones RAL, Ryan AJ, Gough T, Vafabakhsh R, Golestanian R 2007 Self-motile colloidal particles: from directed propulsion to random walk. Phys. Rev. Lett. 99, 048102 (10.1103/PhysRevLett.99.048102) 17678409

[55] Garcia-Pichel F 1989 Rapid bacterial swimming measured in swarming cells of Thiovulum majus. J. Bacteriol. 171, 3560–3563. (10.1128/jb.171.6.3560-3563.1989) 2498293 PMC210087

[56] Vaituzis Z, Doetsch RN 1969 Motility tracks: technique for quantitative study of bacterial movement. Appl. Microbiol. 17, 584–588. 4977222 10.1128/am.17.4.584-588.1969 PMC377747

[57] Schlegel HG 1985 Allgemeine mikrobiologie. Stuttgart, Germany: Georg Thieme Verlag.

[58] Weinstock MT, Hesek ED, Wilson CM, Gibson DG 2016 Vibrio natriegens as a fast-growing host for molecular biology. Nat. Methods 13, 849–851. (10.1038/nmeth.3970) 27571549

[59] Herzog B, Wirth R 2012 Swimming behavior of selected species of Archaea. Appl. Environ. Microbiol. 78, 1670–1674. (10.1128/AEM.06723-11) 22247169 PMC3298134

[60] Brennen AC, Winet H 1977 Fluid mechanics of propulsion by cilia and flagella. Annu. Rev. Fluid Mech. 9, 339–398. (10.1146/annurev.fl.09.010177.002011)

[61] Sleigh MA, Blake JR 1977 Methods of ciliary propulsion and their size limitations. In Scale effects in animal locomotion (ed. Pedley TJ), pp. 234–236. New York: NY: Academic Press, Inc.

[62] Hahm JH, Kim S, Diloreto R, Shi C, Lee SJV, Murphy CT, Nam HG 2015 C. elegans maximum velocity correlates with healthspan and is maintained in worms with an insulin receptor mutation. Nat. Commun. 6, 8919 (10.1038/ncomms9919) 26586186 PMC4656132

[63] Nicolau DV Jr et al. 2016 Reply to Einarsson: The computational power of parallel network exploration with many bioagents. Proc. Natl Acad. Sci. USA 113, E3188 (10.1073/pnas.1605214113) 27226290 PMC4988593

[64] Potsaid B, Bellouard Y, Wen JT 2005 Adaptive Scanning Optical Microscope (ASOM): a multidisciplinary optical microscope design for large field of view and high resolution imaging. Opt. Express 13, 6504–6518. (10.1364/OPEX.13.006504) 19498666

[65] Einarsson J 2016 New biological device not faster than regular computer. Proc. Natl Acad. Sci. USA 113, E3187 (10.1073/pnas.1603944113) 27226291 PMC4988569

[66] Pisinger D 1999 Linear time algorithms for knapsack problems with bounded weights. J. Algorithms 33, 1–14. (10.1006/jagm.1999.1034)

[67] Mckee SA 2004 Reflections on the memory wall In Proc. 1st Conf. on Computing Frontiers, Ischia, Italy, 14–16 April 2004, p. 162. New York, NY: ACM (10.1145/977091.977115)

[68] DOE/NNSA. 2014 Preliminary conceptual design for an exascale computing initiative. Washington, DC: US Department of Energy Office of Science and National Nuclear Security Administration. See https://www.science.energy.gov/~/media/ascr/ascac/pdf/meetings/20141121/Exascale_Preliminary_Plan_V11_sb03c.pdf.

[69] King J, Yarkoni S, Raymond J, Ozdan I, King AD, Mohammadi Nevisi M, Hilton JP, Mcgeoch CC 2017 Quantum annealing amid local ruggedness and global frustration. D-Wave Technical Report Series. 2017(14-1003A-C).

[70] Mitchell JG 2002 The energetics and scaling of search strategies in bacteria. Am. Nat. 160, 727–740. (10.1086/343874) 18707461

[71] Mitchell JG, Kogure K 2006 Bacterial motility: links to the environment and a driving force for microbial physics. FEMS Microbiol. Ecol. 55, 3–16. (10.1111/j.1574-6941.2005.00003.x) 16420610

[72] Landauer R 2000 Irreversibility and heat generation in the computing process. IBM J. Res. Dev. 44, 261–269. (10.1147/rd.441.0261)

[73] Strohmaier E, Dongarra J, Simon H, Meuer M 2017 Top 500 Supercomputers. See https://www.top500.org/lists/top500/.

[74] Yin X, Li F, Bo X, Luo Z, Zuo X 2017 Computation in chemistry: a summary of the development and models of DNA computing. Prog. Chem. 29, 1297–1315.

[75] Lard M, Ten Siethoff L, Generosi J, Månsson A, Linke H 2013 Molecular motor transport through hollow nanowires. Nano Lett. 14, 3041–3046. (10.1021/nl404714b) 24874101

[76] Balaz M, Månsson A 2005 Detection of small differences in actomyosin function using actin labeled with different phalloidin conjugates. Anal. Biochem. 338, 224–236. (10.1016/j.ab.2004.12.012) 15745742

[77] Lard M, Ten Siethoff L, Månsson A, Linke H 2013 Tracking actomyosin at fluorescence check points. Sci. Rep. 3, 1092 (10.1038/srep01092) 23346350 PMC3549537

[78] Ooms MD, Dinh CT, Sargent EH, Sinton D 2016 Photon management for augmented photosynthesis. Nat. Commun. 7, 12699 (10.1038/ncomms12699) 27581187 PMC5025804

