# Supplementary material for: Something has to give: scaling combinatorial computing by biological agents exploring physical networks encoding NP-complete problems
Source: Interface Focus. 2018 Oct 19;8(6):20180034. doi: 10.1098/rsfs.2018.0034 (PMC6227808; doi:10.1098/rsfs.2018.0034)
Supplement: Supplementary Information [file rsfs20180034supp1.docx]

**- Supplementary Information –**

**Interface Focus**

**Something has to give: Scaling combinatorial computing by biological agents exploring physical networks encoding NP-complete problems**

Falco C.M.J.M. van Delft, Giulia Ipolitti, Dan V. Nicolau Jr., Ayyappasamy Sudalaiyadum Perumal, Ondrej Kaspar ,Sara Kheireddine, Sebastian Wachsmann-Hogiu, Dan V. Nicolau

**SI-1 Mathematical formulation of network encoding**

The ‘hardware’ component of the computational network-based devices, i.e., networks whose design reflects a particular problem of interest, could be mathematically formulated as follows. An arbitrary decision, i.e., yes/no, problem (D) whose instances, i.e., specific cases of the problem, are denoted as D_1_, D_2_, … can be represented by a class of networks of nodes and connections, E, that captures the structure of the decision problem. This representation is made in such a way that, for every instance D_i_ of D, one can find a directed graph, E_i_, in which a path from any of a predefined set of input nodes to any of a predefined set of output nodes exists if — and only if — the decision problem on D_i_ (as defined above) has an answer in the affirmative. Conversely, we need to guarantee that such a path cannot be found if the decision problem on D_i_ has a negative answer. If the NP-Hardness Assumption is true, then the number of unique paths through the networks encoding the instances will, in the worst case, grow exponentially with instance size, thus necessitating the use of a number of agents larger than this number of paths, in order to comprehensively explore the solution space. Consequently, the engineering future of computing with networks rests on finding a physical implementation strategy that is able to marshal potentially very large numbers of agents.

**SI-2 Nomogram for Field-of-View and Cardinality in case all agents move**
**through the network channels in singular queues (no overtaking).**

The expected chip size is shown in the nomogram in Figure SI-1 (left hand side) as a function of the channel widths (dependent on the agent sizes) for the “prime number” SSP devices for various cardinalities. The horizontal black dashed lines delimit the sizes of 4, 6 and 8 inch silicon wafers – the standards in the semiconductor industry. The vertical yellow bars indicate the line widths for devices running molecular motors-driven cytoskeletal filaments, i.e., actin filaments, microtubules, as well as small (*E. coli*) and large (*E. viridis*) microorganisms. As examples, the green, red and black arrows indicate the chip sizes of, respectively, the cardinality 15 network for microtubules, the cardinality 5 network for *E. coli*, and the cardinality 5 network for *E. viridis*.

Because of the competition between resolution and the FoV, the whole imaging of the computing area requires the employment of the maximum useable pixel size (MUPS) that can still discriminate the individual agents, as well as the legal (and illegal) turns they take. Regarding the latter, the middle panel in Figure SI-1 presents the SSP chip split junction design with a given channel width (top). Also, the split junction design is shown with an overlay of pixels 5 times larger than the channel width, which is the limit that would still allow the system to follow vertical traffic (in blue), diagonal traffic (in yellow), and whether the splits are being used for changing traffic direction (in purple), or if the central pass crossing (in green) is used. The legal pass and split traffic is indicated by the groups of three squares underneath the overlaid design. A typical example of an illegal turn that would be detectable is yellow-green-blue. Apart from being 5 times the channel width, the MUPS value should also allow discernment of the individual agents. Hence, the MUPS value should also be smaller than the agent length.

The right-hand side of Figure SI-1 represents the square root of FoV versus the MUPS value. The black crosses indicate the intersection of the largest attainable FoV (as a square root) with the minimum attainable MUPS for various optical imaging technologies, i.e. their resolution limit. The useable optical range is obtained by the intersection of sqrt(FoV)-MUPS range with the diagonal black line indicated as ‘Unity’. At the point where the top horizontal border meets the ‘Unity’ line, the MUPS value is equal to the total FoV, meaning that only one pixel fits in the frame, obviously far from any reasonable application. To fully exploit the frame size available, the MUPS value should be as close as possible to the resolution limit. The vertical blue bars indicate the MUPS for devices running molecular motors-driven filaments, and small-and large microorganisms. Similarly to the left panel in Figure SI-1, the horizontal green, red and black arrows copy the chip sizes of respectively the cardinality 15 network for microtubules, the cardinality 5 network for *E. coli*, and the cardinality 5 network for *E. viridis*. If the spot where the vertical green, red, or black arrows, respectively, meet their equivalent horizontal arrows is inside a ‘technologically-achievable’ sqrt(FoV)-MUPS triangular area, the corresponding optical technique is, in principle, useable for monitoring the computation process using a single FoV. It follows that a cardinality 5 network for *E. viridis* can be monitored by a macro-lens equipped camera, whereas the cardinality 5 network for *E. coli* and the cardinality 15 network for microtubules can be monitored by a lens-less microscope.

**Note that this scenario assumes that there are no agents overtaking each other in the channels either laterally or by crawling over each other.**


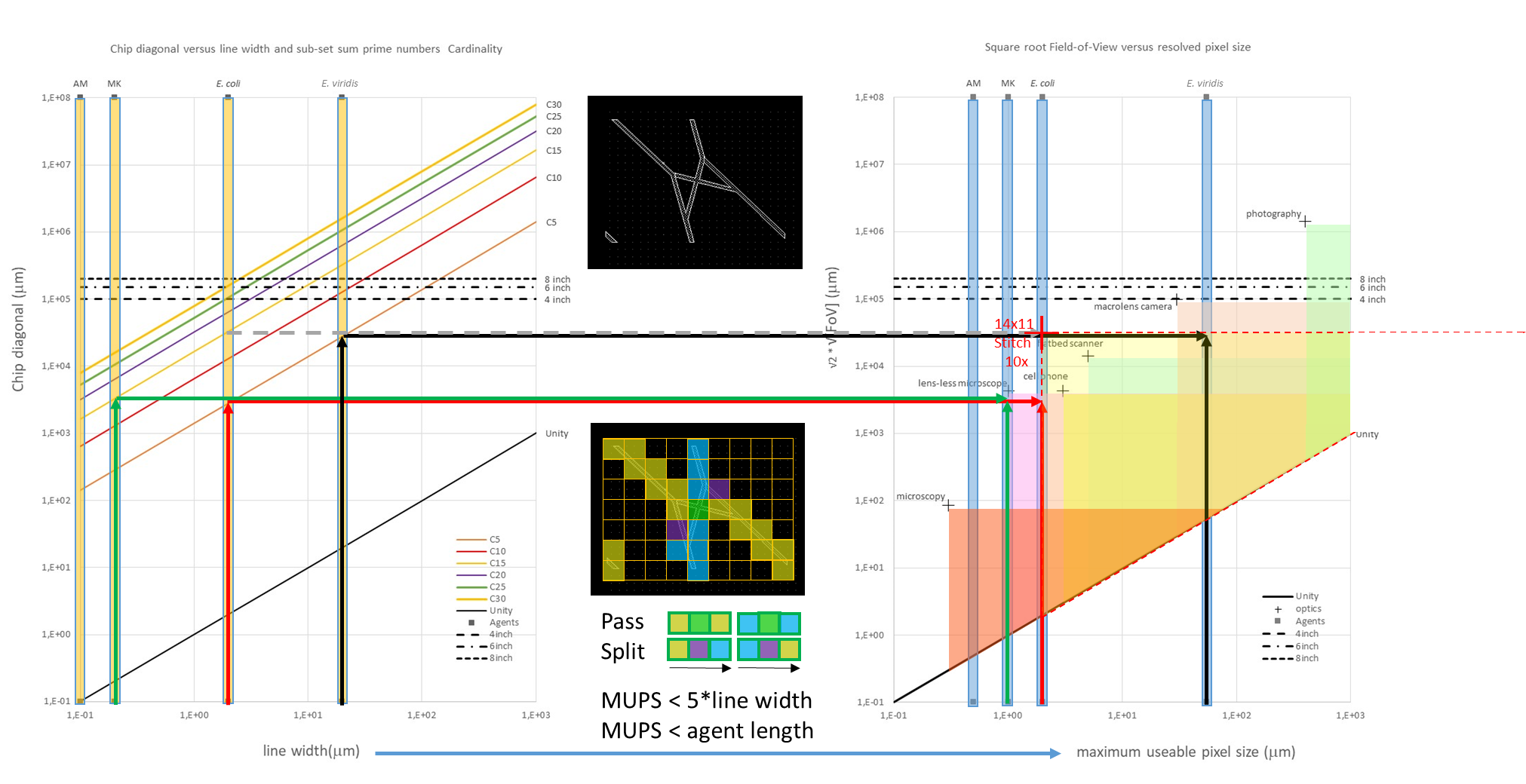


Figure SI-1 Nomogram for network size versus line width and cardinality,

and Field-of-View versus Maximum Useable Pixel Size and resolution.

Also shown is an example of an enlarged work window by image stitching
(red cross); the limits of this method are discussed briefly in the main text and in detail in SI-3.

**SI-3 Stitching FoV’s while keeping track of all agents**

When the area to be imaged exceeds the Field-of-View (FoV) of the imaging system, image stitching can be performed to ‘bridge the gap’, although not without some loss of information. Of course, this loss can be minimised through faster switching speeds, which in turn are limited by the mechanical capabilities of the microscope stage. To perform these calculations, the following factors should be taken into account:

- Speed of biological agent used
- Exposure time of each partition of the image

For the sake of simplifying our sample calculations, we are assuming no horizontal or vertical image overlap, negligible exposure times (which in reality can go as low as 1-10 ms for bright field imaging and as high as 500 ms for fluorescence imaging), perfect functioning of pass and split junctions in our subset sum networks (SSN), and unidirectional movement of our biological agents with no U-turns. Factoring in the density of the agents necessitates choosing the optimal switching speed based on dependency on body length in case of high agent density, and based on distance between adjacent junctions in case of low agent density.

For the scenarios described below, numerical examples are shown for E. *coli* K-12, where body width (BW) = 0.5 μm, body length (BL) = 2.5 μm, and speed v = 10 μm/s. Vertical and Diagonal Junction Distances (i.e. between two adjacent junctions) for the subset sum problem network (SSP) fabricated with 2 um-width channels for E. *coli*, are 60 μm (VJD) and 100 μm (DJD), respectively.

- In the case of high agent density (scenario where **more accurate** tracking is required)

1. Speed of agent v = 10 μm/s
2. Speed v_b_ of agent in body lenghts/s: v_b_ = v / BL = 4 bl/s
3. Time period to return to original image partition (in case we wish the agent to have moved only ½ a body length) t_r_ = 0.5*BL/v = 0.5bl / v_b_ = 125 ms
4. The average switching time between partitions t_s_ is determined by t_s_ = t_r_ / n*m _,_ where n and m are the number of partitions in x and y respectively (e.g. 2x2, 3x4). Hence, for a 2x2 stitching mode, t_s_ = 125ms / 4 = 31.25ms

- In case of low agent density (scenario where **less accurate** tracking is acceptable)

1. Speed of agent v = 10 μm/s
2. Speed of agent (v_v_ or v_d_) in vertical- or diagonal junction steps per second
   v_v_ = v / VJD = 0.17 vjs/s and v_d_ = v / DJD =0.1 djs/s
3. Time period to return to original image partition

(in case we want the agent to have moved by 1 vjs or 1 djs)

t_v_ = VJD / v = 1vjs/ v_v_ = 6s and t_d_ = DJD / v = 1djs/ v_d_ = 10s

1. The average vertical switching time between partitions t_sv_ is given by t_sv_ = t_v_ / n*m _,_ where n and m are the number of partitions in x and y respectively (e.g. 2x2, 3x4);

the average diagonal switching time between partitions t_sd_ is given by t_sd_ = t_d_ / n*m _._

Hence, for a 2x2 stitching mode t_sv_ = 6s/4 = 1.5s and t_sd_ = 10s/4 = 2.5s .

The calculations shown above describe two extreme cases in terms of agent density, and as a result the necessary corresponding tracking accuracy. However, in real-life situations, we will mostly have intermediate scenarios that fall between both, and it would be up to our discretion to choose the appropriate values to use.

As such, the overall parametric equation to calculate switching time (t_s_) can be described as:

$t_{s}$ = $\frac{t_{r}}{nm}$ = $\frac{d}{v^{*} nm}$

where *t_s_* is the switching time between image partitions, *t_r_* is the time period to return to original image partition, *n* is the number of image partitions in the form ‘n x m’, *d* is the displacement we would like our agent to have moved by the time we return to the original image partition expressed in terms of BL/VJD/DJD etc., and *v^*^* is the agent speed expressed in terms of BL/VJD/DJD etc. rather than in unit length per second.

The switching time (t_s_) encompasses two values, namely translation time (t_t_) and exposure time (t_e_), where t_t_ can be expressed as:

$t_{t}$ = $\frac{\mathrm{FoV}}{v_{ss}}$

where *FoV* is the diagonal field-of-view of the system, and *v_ss_* is the speed of the scanning microscope stage. Moreover, t_e_ is dependent on illumination technique (e.g. bright field), system magnification (inversely proportional), and when using fluorescence, the fluorophore used. It follows from this that t_s_ $\geq$ t_t_ + t_e_ in order to achieve the required agent tracking accuracy.

In terms of what the current technology can offer, the fastest motorised translational stage, to the best of our knowledge, offers a maximum speed of 250 mm/s, with an accuracy < 3 um. This stage utilises a servo motor as its actuator, and is available from Thorlabs Inc.

Once again, using two extreme scenarios for the FoV can help shed light on what this system could help us achieve. If we use a 100x objective (NA=1.4), which gives us a FoV ~ 60 um, we get t_t_ = 240 us. When pairing this with a reasonably low t_e_, one can achieve high accuracy with a large number of partitions, and a high resolution (~ 0.5-1 um), but the overall FoV covered would be quite small. On the other hand, if we use a 2x objective (NA=0.3), with FoV ~ 1.5 cm, we get t_t_ = 60 ms. Based on our previous calculations, regardless of the t_e_ used, we will not be able to use this system to scan even the smallest (2x2) partition matrix for the most accurate tracking scenario (t_s_ <= 31.25 ms). However, we can still use this for a slightly less accurate tracking scenario while covering a larger overall FoV with a fairly good resolution (~ 3-5 um).

Typically, for observing E. *coli* in the SSN, we use a 4x objective (NA=0.16) under fluorescence, with t_e_ = 500 ms. In order to observe the largest chip we have with cardinality 30 (C30), which has an area of ~ 13x10 cm, and using the same 4x objective with FoV ~ 1 cm, we would need 13x10 partitions. For this set-up, and using the more accurate tracking approach, we would need a t_s_ = [125 ms / (13*10)] = 0.962 us = ~ 1 ms, which is unattainable given the large t_e_ requirement. Moreover, using the less accurate tracking approach, we would need a t_s_ = [10 s / (13*10)] = ~ 0.077 s = 77 ms, which once again is unfeasible. If, however, we disregard t_e_, we would need a $v_{ss}$ = 10 m/s, and $v_{ss}$ = ~ 13 cm/s respectively. Since the SSN has a triangular profile, and only half of the rectangular partition grid will be traversed by the moving stage, the calculated t_s_ should be multiplied by 2, which would result in half the speed requirement. This, even with the improved requirements, is nowhere near feasible given the current available technology. While ignoring t_e_ is unrealistic for tracking E. *coli*, it could potentially work for bacterial species that provide good contrast, such as cocci.

Table SI-I shows which chips can be observed with image stitching using the specific objectives available on an Olympus IX83 microscope. This comparison was made using appropriate exposure times for each magnification, in addition to the translation time using the stage speed available (13 mm/s).

It shows that -with stitching- up to C15 can be observed with a 10x objective and up to C20 with a 4x objective, compared to C4 and C11 respectively without stitching (i.e. one FoV). However, just to relax to speed requirements and other elements that were not taken into account (such as areas of image overlap, precision issues, etc.), the limit will be more likely at C15 when using the 4x objective.

**Table SI-I**

**Objective NA Resolution FoV Cardinality Stitching Cardinality**

**(μm)** **(μm) 1 FoV limits Stitched FoV**

2x 0.3 5 15000 11 4 x 3 21

4x 0.16 3 10000 9 6 x 4 20

10x 0.4 2 2000 4 14 x 11 15

20x 0.75 2 1000 3 24 x 18 14

40x 0.96 2 500 27 x 20 11

60x 1.35 1 200 32 x 24 8

100x 1.4 0.5 60 39 x 30 5

**SI-4 Method 1: Sub-set Sum Problem calculation by various electronic chips**

A computer program to solve the Subset Sum Problem was developed in the C programming language to enable low-level memory access, efficient mapping to machine instructions and flexibility. Out of several algorithms to solve the SSP, a naïve approach was adopted to emulate the Biological Chip logic and operations. Therefore, it was possible to establish a comparison between electronic and biological devices in terms of performance times with increasing size of the problem (Cardinality).

The SSP algorithm was designed to recursively explore all $N$-elements in the set and to log the $k$ winning combinations according to the $N$ chooses $k$ subsets $\sum_{0\leq k\leq N} \binom{N}{k}=2^{N}$. Its running time falls in the order ${O(2}^{N}N)$ due to the SSP combinatorial nature and the highest computed sum of $N$-elements to identify subsets. Similarly, the bio-device was designed to find all possible $k$ subsets sums by exploring $2^{N}$possible combinations, times the $N$-elements considered for solution identification.

The algorithmic naïve approach, combined to its lack of any pre-existing knowledge and bias of problem clauses, enabled us to benchmark the Biological chip performance analysis with performances of historical Intel processors. Being RAM Memory and Clock Speed the major factors affecting CPU speed, we replicated computing resources of Intel382 SX and DX, Intel486 DX, Intel DX2, Intel Pentium and Intel Core Quad 2Processor versions by simulating part of their computer hardware with Virtual Machines. Thus, we were able to scale down RAM and clock speed and record performance times on SSP.

**SI-5 Method 2: Simulation of Sub-set Sum Problem calculation**
 **by a hypothetical biological chip**

The performance of electronic computers was compared to hypothetical bio-computer performance simulated for the following agents: *E. coli*, *V. natriegens*, *M. janaschii*, and *M. villosus*, as well as Microtubules and Actin filaments. For including cell division in the simulation of the bacterial species used, the following doubling times have been used: *M. janaschii*: 74 min., *M. villosus*: 45 min., *E. coli*: 30 min., and *V. natriegens*: 15 min.

The independent and parallel behaviour of a large number of self-propelled biological agents was reproduced and scaled taking into consideration various factors: network and channels geometry varying with increasing SSP size, agents’ random walk behaviour over network exploration and physical rules of motion and logic given by network structures and agents sizes, speed and characteristic behaviour.

**SI-6 Electronic reference device**

By ‘reverse engineering’ starting from the pass- and split junctions for the sub-set sum bio-computation device described in this paper, an electronic equivalent device was designed. Originally, an analog circuit was conceived, but simulations showed that already with a limited number of nodes, leakage currents would render the readout of such a device unreliable. Hence, a digital electronic demo device (shown in Figure SI-2) with LED readout and IC HEF4019BP containing AND and OR gates (shown in Figure SI-3) was constructed; this particular DEMO device has a total sum of 6 only, but it has flexible nodes, which can be switched from pass- to split junctions by activating bus lines.


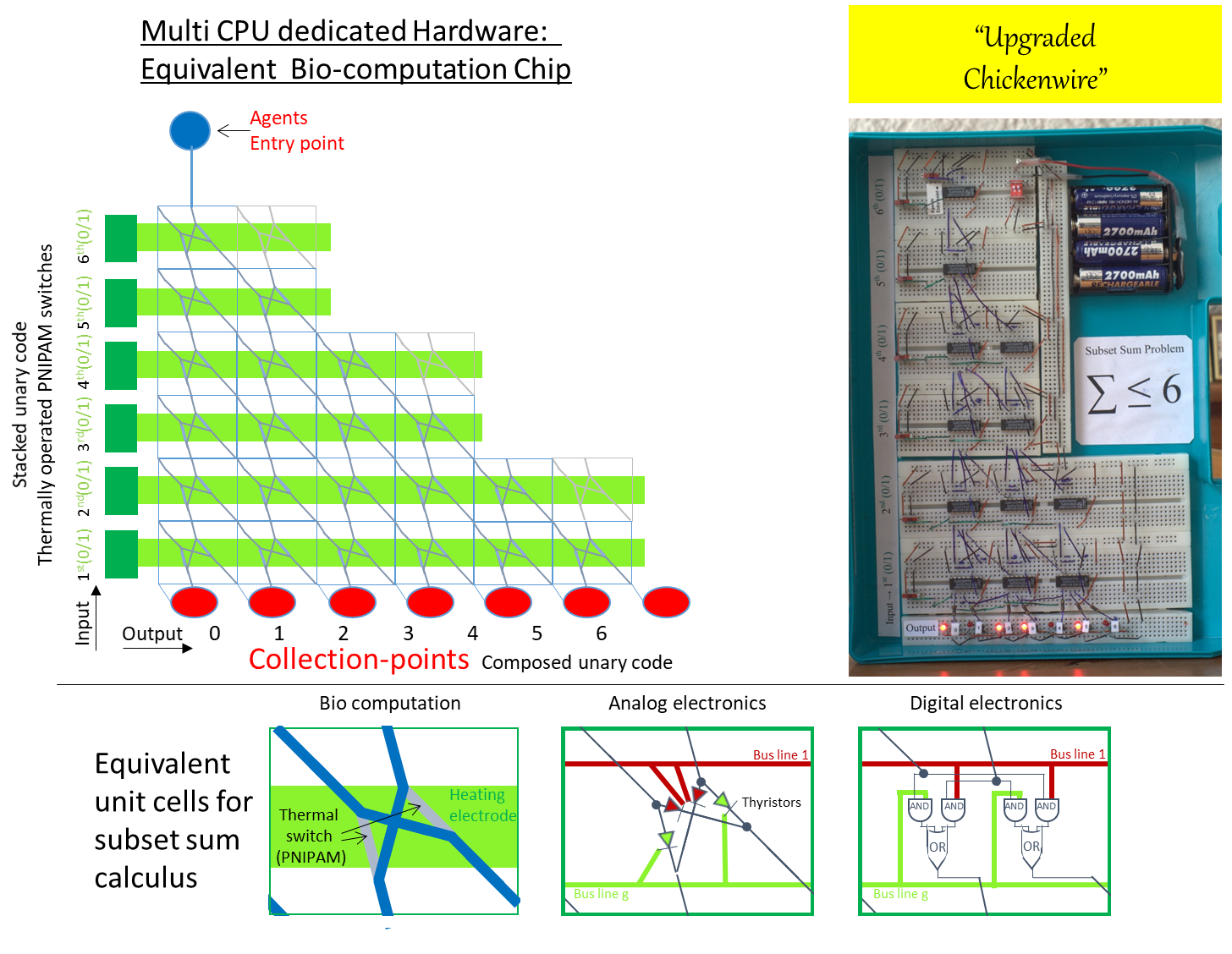


Figure SI-2 Electronic demo device - Operation Upgraded CHicken wire (OUCH!)


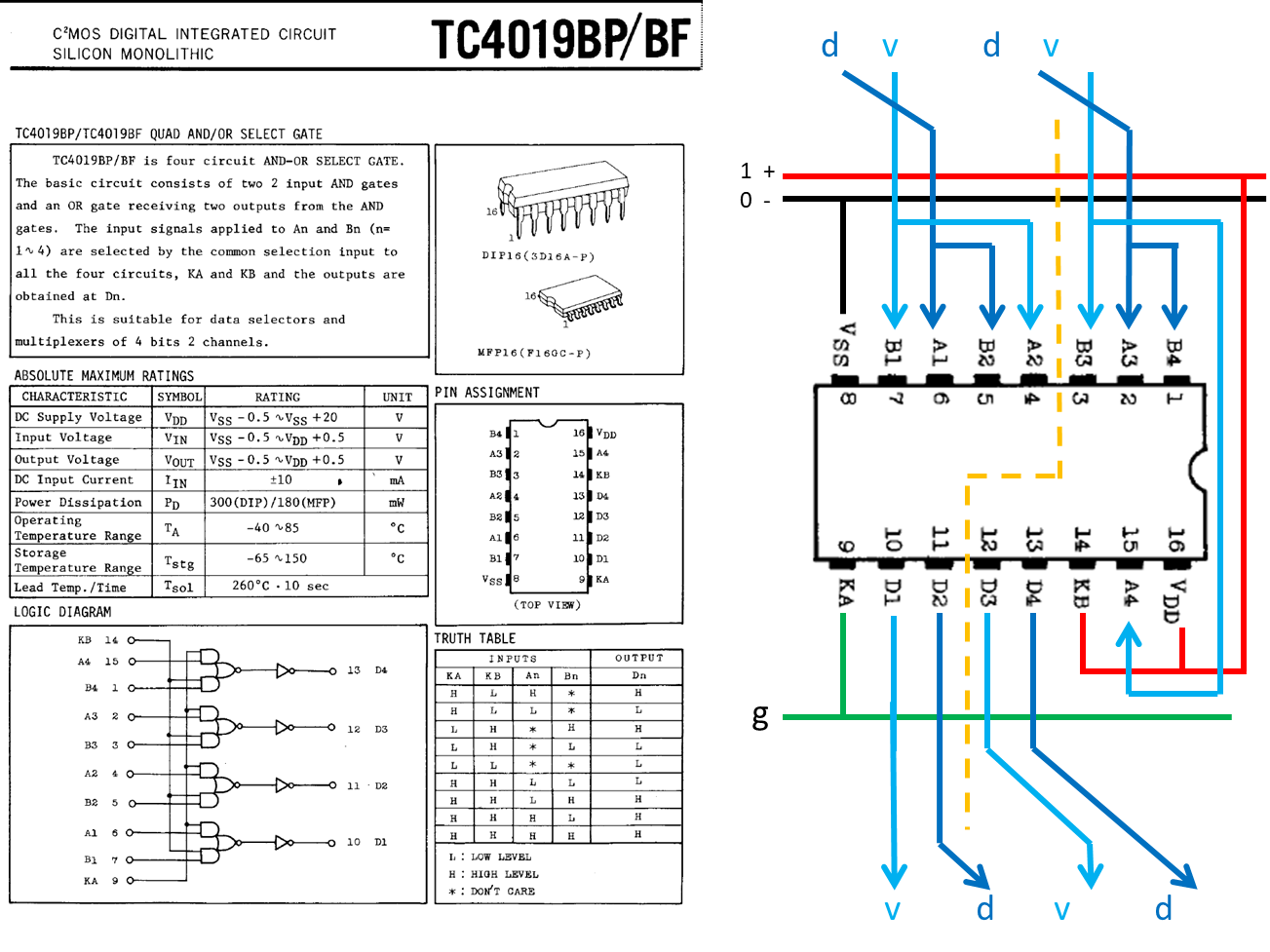


Figure SI-3 IC HEF4019BP
Note that this IC can accommodate
two pass- or two split junctions simultaneously

<http://www.datasheet4u.com/> search for TC4019BP

<http://www.datasheet4u.com/datasheet-pdf/Toshiba/TC4019BP/pdf.php?id=530848>
